# Evolving stability and pH-dependent activity of the high redox potential *Botrytis aclada* laccase for enzymatic fuel cells

**DOI:** 10.1038/s41598-017-13734-0

**Published:** 2017-10-20

**Authors:** Stefan Scheiblbrandner, Erik Breslmayr, Florian Csarman, Regina Paukner, Johannes Führer, Peter L. Herzog, Sergey V. Shleev, Evgeny M. Osipov, Tamara V. Tikhonova, Vladimir O. Popov, Dietmar Haltrich, Roland Ludwig, Roman Kittl

**Affiliations:** 1 Department of Food Sciences and Technology, VIBT – Vienna Institute of BioTechnology, BOKU – University of Natural Resources and Life Sciences, A-1190 Vienna, Austria; 2 Biomedical Sciences, Health and Society, Malmö University, 20560 Malmö, Sweden; 3 Bach Institute of Biochemistry, Research Center of Biotechnology RAS, 119071 Moscow, Russian Federation

## Abstract

Fungal high redox potential laccases are proposed as cathodic biocatalysts in implantable enzymatic fuel cells to generate high cell voltages. Their application is limited mainly through their acidic pH optimum and chloride inhibition. This work investigates evolutionary and engineering strategies to increase the pH optimum of a chloride-tolerant, high redox potential laccase from the ascomycete *Botrytis aclada*. The laccase was subjected to two rounds of directed evolution and the clones screened for increased stability and activity at pH 6.5. Beneficial mutation sites were investigated by semi-rational and combinatorial mutagenesis. Fourteen variants were characterised in detail to evaluate changes of the kinetic constants. Mutations increasing thermostability were distributed over the entire structure. Among them, T383I showed a 2.6-fold increased half-life by preventing the loss of the T2 copper through unfolding of a loop. Mutations affecting the pH-dependence cluster around the T1 copper and categorise in three types of altered pH profiles: pH-type I changes the monotonic decreasing pH profile into a bell-shaped profile, pH-type II describes increased specific activity below pH 6.5, and pH-type III increased specific activity above pH 6.5. Specific activities of the best variants were up to 5-fold higher (13 U mg^−1^) than *Ba*L WT at pH 7.5.

## Introduction

Laccases (EC 1.10.3.2, CAZy AA1), alongside ascorbate oxidase and bilirubin oxidase, belong to the group of three-domain multicopper blue oxidases^1^. This group of enzymes binds four copper atoms as cofactors in two active sites. The type I copper centre (T1) is situated in a surface exposed cleft and oxidises phenols, aromatic or aliphatic amines^2^ corresponding to its *in vivo* role in fungal pigment formation, lignin degradation, or detoxification^3^. The second active site consists of a type II/type III trinuclear copper cluster (T2/T3) located in the core of the protein. The electrons obtained from substrate oxidation are transferred via intramolecular electron transfer from the T1 to the trinuclear copper cluster. Here, a dioxygen molecule bound between the two T3 copper ions acts as final electron acceptor and is fully reduced to two water molecules by a four-electron reduction^4^.

For the oxidation of recalcitrant substrates like lignin compounds a high redox potential is necessary for fungal laccases. Therefore, laccases exhibiting a high T1 redox potential of 730–790 mV *vs*. SHE are typically found in white-rot basidiomycetes, whereas for ascomycete and other basidiomycete laccases lower T1 redox potentials of 470–710 mV *vs*. SHE and for bacterial and plant laccases even lower potentials of 340–490 mV *vs*. SHE are reported^5,6^. The laccases from *Botrytis aclada* (*Ba*L) and *Botrytis cinerea* (*Bc*L) are the currently only known ascomycete high redox potential laccases (HRPLs) (*Ba*L, 720 mV^7^; *Bc*L, 780 mV *vs*. SHE^8^). *Ba*L also shows low inhibition by chloride^9^; a feature typical for lower redox potential ascomycete laccases rather than for basidiomycete HRPLs, which are strongly inhibited by halides (i.e. *I*_50, NaCl_ = 40 mM of *T*. *versicolor* laccase)^10^. Major goals for the engineering of laccase towards an efficient cathode biocatalyst in implantable biomedical devices, i.e. an enzymatic fuel cell, are sufficient catalytic turnover at physiological pH, high T1 redox potential, low inhibition by chloride ions^11^, and high longevity.

Therefore, engineering of HRPLs focused on higher thermostability^12,13^, a reduced inhibition by halides^14^, and increased activity at neutral pH^13,15–17^. The most recent work to improve a HRPL in this direction by enzyme engineering was performed by Mate and co-workers^6,14,18–20^. They created a variant from the unclassified basidiomycete PM1 for functional expression in *S*. *cerevisiae* by eight rounds of directed evolution^18,20^, rendered it active in blood by four further evolutionary rounds^19^ and functionally expressed it in *P*. *pastoris* ^6,14^. Conclusions from these studies are that mutations influencing activity pH dependence very likely also influence redox potential and the kinetics of the enzyme.

The properties of *Ba*L (highest reported chloride tolerance (*I*_50_, _NaCl_, _DMP_ = 1.4 M)^9,14^, high expression levels and reasonable specific activities at pH 6.0 of 14.1 and 35 U mg^−1^ for DMP and ABTS, respectively^9^) make it a promising candidate for the application as a biocathodic element in an implantable enzymatic fuel cell and a good candidate for protein engineering. To the best of the authors knowledge, none of the reported HRPLs reach chloride tolerance levels comparable to the wild-type HRPL from *Botrytis aclada* (*Ba*L WT) investigated in this work^9^. To improve *Ba*Ls performance at physiological conditions (37 °C, pH 7.4) an increase of activity at neutral pH and of the longevity is essential.

In this work, we present the combination of evolutionary enzyme optimization and structure-based engineering towards enhanced stability and activity at elevated pH of a chloride tolerant HRPL for the application in enzymatic biofuel cells. In a first step, a simple and robust high-throughput differential screening assay to handle large mutant libraries was used. In a second step *Ba*L variants were identified from screened directed evolution and site-saturation mutagenesis libraries. Finally, the best performing mutations were selected and combined into multiple variants. A detailed biochemical study of 14 *Ba*L variants was performed to characterise the mutations and evaluate their effect on thermostability, pH profile, and kinetic constants.

## Results and Discussion

### Generation of laccase variants

#### Directed evolution

**Figure 1:**
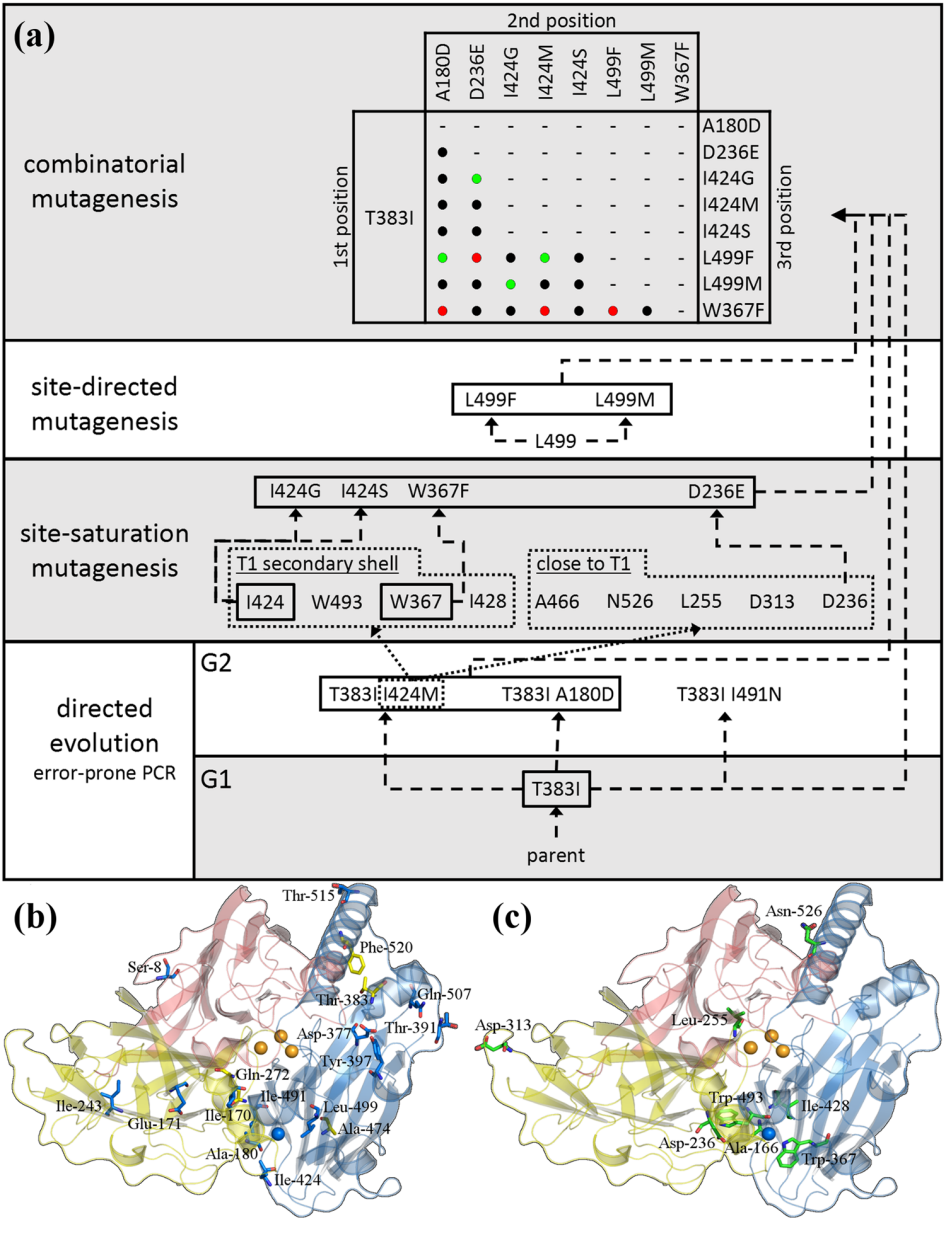
Generation of laccase variants (**a**) and cluster of mutations (**b**, **c**). The scheme in (**a**) shows from bottom to top in a chronological order the applied mutagenic technique and the obtained variants. Directed evolution round 1 (G1) yielded the thermostable variant T383I, while G2 yielded the three indicated double-variants with higher relative activity at elevated pH. Dashed arrows indicate the direct creation of the respective variant, while dotted arrows (I424M to site-saturation mutagenesis) mean that the location of I424 in the crystal structure was the motivation to target the indicated residues in vicinity to the T1 copper. Variants edged in a black lined box were beneficial and used for combinatorial mutagenesis. Green, black and red dots in the combinatorial mutagenesis matrix indicate the created triple variants showing increased activity at pH 6.5, activity at all and no activity at all, respectively. The images below show residues subjected to a mutation found in (**b**) directed evolution (blue residues, affecting pH/activity relationship; yellow residues, affecting thermal stability) and (**c**) site-saturated mutagenesis experiments (residues in green). The copper ions are shown as spheres (blue, type-I copper; orange, trinuclear copper cluster). The cupredoxin-like domains I, II and III of *Botrytis aclada* laccase (PDB entry: 3SQR) are colored in red, yellow and blue, respectively.

*B*. *aclada* laccase shows distinct pH optima in the acidic region for various substrates. Oxidation of 2,2′-azino-bis(3-ethylbenzothiazoline-6-sulfonic acid) (ABTS) and 2,6-dimethoxyphenol (DMP) resulted in monotonously decreasing pH profiles with optima lower than pH 3.0; other small phenolic substrates such as guaiacol or hydroquinone exhibited narrow pH optima around pH 4.0^9^, while its activity in the neutral region is significantly reduced^9^. Furthermore, it is only moderately thermostable with a half-life of <5 min at 55 °C. The main objectives of the screening and selection of a mutational library therefore were the identification of variants showing increased activity at neutral pH as well as increased thermostability. Random mutagenic plasmid libraries were created for two rounds of selection. The libraries comprised 148,500 and 82,900 plasmid-carrying *E*. *coli* clones in the first and second round, respectively. After transformation of the plasmid libraries into *P*. *pastoris*, a total of 38,800 and 33,400 transformants were pre-screened on agar plates for secreted laccase activity. In both rounds about 20% of the transformants expressed active laccase, and were subjected to a differential activity screening (comparison of activity assays at pH 5.0 and 6.5; additional incubation at 55 °C for 30 min, Supplemental Fig. S1) in 96-well plates. Approximately 1.7% and 3.5% of the transformants from the first and second round, respectively, showed a higher activity at elevated pH compared to *Ba*L WT, while less than 0.5% of the transformants retained significant activity after incubation at 55 °C. The most promising variants obtained were the thermostable variant T383I identified in the first screening, and the variants T383I/A180D, T383I/I424M and T383I/I491N from the second generation with a 1.5-, 2.2- and 2.6-fold higher relative activity, respectively, at pH 6.5 with DMP (Fig. 1a, Supplemental Table S1, S7, and S9).

#### Site-saturation mutagenesis

According to the crystal structure of *Ba*L (PDB entry: 3SQR), the mutation I424M, which increases enzymatic activity at neutral pH, is found in the secondary shell of amino acids embracing the T1 copper within a distance of 5–7 Å. To find other possibly effective mutations in this region, residues within the secondary shell and additional positions close to T1 (Fig. 1b,c) were targeted by site-saturation mutagenesis. This selection of residues considers findings of Maté and co-workers on the evolution of basidiomycetous laccases and the variants OB-1^18^ and Chu-B^19^. A site-saturation plasmid library was created for each of these nine positions. Each library contained at least 5000 plasmid-carrying *E*. *coli* clones, from which 400 *P*. *pastoris* transformants were generated and subjected to the differential screening assay. Mutations exhibiting higher activity at neutral pH relative to *Ba*L WT were identified in three of the nine targeted amino acid positions (D236E, W367F and I424G).

#### Site-directed mutagenesis

The function of the non-coordinating axial T1 copper ligand L499 in *Ba*L was previously studied^21–24^. The variant L499M was partially characterised earlier and showed an altered pH behaviour^7^. Therefore, this variant was included in subsequent combinatorial mutagenesis studies. Furthermore, we also created the variant L499F based on the observation that fungal laccases either show Leu or Phe in this position^25,26^.

#### Combinatorial mutagenesis

A total of 24 triple variants were created by combining mutations selected from directed evolution, site-saturation, and site-directed mutagenesis experiments (Fig. 1). All variants include the thermostability-improving mutation T383I and vary in their second and third mutation to investigate the effects of the combined mutations regarding improved enzymatic activity at neutral pH. To select clones for subsequent laccase production, the laccase activity of 15 clones from each triple variant was first visually evaluated on BMG agar plates containing ABTS via the diameter and intensity of the green halos formed upon ABTS oxidation. The matrix in the top section of Fig. 1a shows the created triple-variants and highlights those exhibiting increased activity and no activity, respectively.

#### Location of mutations

All amino acid positions with beneficial mutations found in this study are indicated in Fig. 1b and c. These mutations cluster around the T1 copper centre and around a rather surface-exposed region in the cupredoxin-like domain III. Mutations affecting the pH behaviour are positioned around or close to the T1 copper binding-site (D236E, I424G and L499F). In contrast, variants with increased thermostability show amino acid exchanges in some distance to the T1 copper centre. The most beneficial mutation with respect to thermostability, T383I, is located in a surface-exposed helix of domain III.

### Increased thermostability

The differential screening approach used for all libraries included a step to evaluate increased thermostability of variants, i.e., a 30-min incubation at 55 °C. Based on this preliminary screening, six clones showed reasonable activity in the supernatant and increased stability (Fig. 1), albeit to a varying extent, retaining 22–65% of their initial activity after incubation. Sequencing unveiled two of the clones as wild-type laccase, two with a T383I-, one with a A474D-, and one with a Q272H- and a F520L-amino acid exchange. *Ba*L variant T383I showed by far the most pronounced stability increase and was characterised in detail. Compared to *Ba*L WT, the half-life time (t_50_) of T383I at 55 °C was increased more than 2.5-fold and the temperature of half-inactivation T_50_, where 50% of the enzyme molecules are inactivated within 10 min, was increased by 6.2 °C from 47.2 to 53.4 °C (Table 1). Similarly, the transition midpoint temperature T_m_ determined by differential scanning calorimetry (DSC) increased by 5.6 °C from 69.0 to 74.6 °C. This stabilizing effect was verified by a PDBePISA calculation (http://www.ebi.ac.uk/pdbe/pisa), which revealed that T383I lowers the solvation energy of the isolated structure by 0.7 kcal mol^−1^ (Supplemental Table S2).

**Table 1:** Temperature stability of *B*. *aclada* laccase and *Ba*L variants.

*variant*	*pH 4*.*0*, *10 min*	*pH 4*.*0*; *55 °C*	Half-life t_50_[min]	Δ to WT	*pH 4*.*5*; *50–90 °C*	Δ to WT
	T_50_, [°C]	Δ to WT			melting temperature	
					T_m_ [°C]	
*Ba*L	47.2	0.0	4.0	0.0	69.0	0.0
T383I	53.4	+6.2	10.5	+6.5	74.6	+5.6
D236E	46.2	−1.0	4.2	+0.2	68.0	−1.0
I424G	45.5	−1.7	3.8	−0.2	70.7	+1.7
L499F	45.1	−2.1	5.3	+1.3	68.2	−0.8

The stability of *Ba*L WT and selected *Ba*L variants in terms of temperature of half-inactivation T_50_, half-life of activity t_50_ and melting temperature T_m_ as well as the difference to *Ba*L WT are shown. The temperature of half-inactivation T_50_ is defined as the temperature in °C at which 50% activity after 10 min incubation is retained. The half-life t_50_ is defined as the time in min after which 50% activity at an incubation at 55 °C is retained. The melting temperature T_m_ is the transition midpoint temperature derived from DSC.

### Shifting laccase activity towards neutral pH

HRPLs are known to have an acidic pH optimum and show almost no residual activity at neutral pH. When using the substrate ABTS, the basidiomycete laccases from *Trametes versicolor*, *Trametes hirsuta*, *Trametes villosa*, *Pleurotus ostreatus* and the unclassified basidiomycete PM1 have their pH optima around pH 3.0 and show no significant activity above pH 6.5–8.0^15,18,27–32^. When employing phenolic substrates like DMP or syringaldazine, the pH optima are found around pH 5.0 but again no activity is observed above pH 7.0–8.0^14,15,18,19,28,32^.

**Figure 2:**
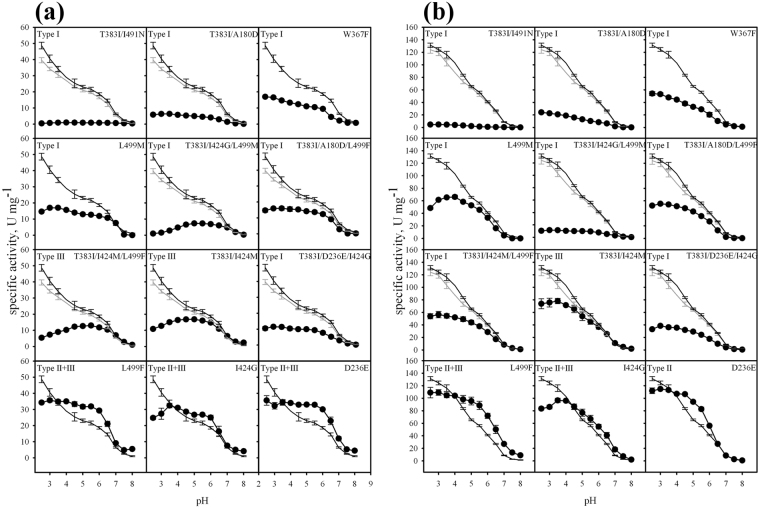
pH-profiles showing specific activities of *Ba*L variants with the substrates 2,6-dimethoxyphenol DMP (**a**) and ABTS (**b**). *Ba*L WT ; T383I ; depicted variant . The pH-type as defined in the text is indicated in each panel: pH-type I, change of monotonically decreasing pH profile to a bell-shaped pH profile through lower specific activity at acidic and not affected activity at less acidic and neutral pH; pH-type II, higher specific activity between pH 3.0 and 6.5; pH-type III, higher specific activities above pH 6.5. Error bars result from at least three independent measurements.

In this study, we screened for enzyme variants showing increased activity at neutral pH using a differential screening approach, in which the ratio of activities with ABTS or DMP at pH 6.5 versus pH 5.0 was calculated. The higher the ratio, the higher the variant was ranked. In that way, different expression levels in the 96-well plate cultivations were compensated for. By using this approach, we could also select variants with shifted pH profiles, but a lower overall specific activity. pH profiles for the substrates DMP and ABTS were measured to identify mutations that change the monotonously decreasing pH profile curve of *Ba*L WT. Three out of 12 variants that showed a higher relative activity at pH 6.5 compared to pH 5.0 (Supplemental Fig. S2) also had higher specific activities than *Ba*L WT at this pH with both substrates DMP and ABTS (Fig. 2, Supplemental Tables S8, S10).

The effects of the mutations on pH profiles for both substrates as shown in the pH vs specific activity curves can be grouped in three types: pH-type I shows a change of the monotonically decreasing pH profile of ABTS to a bell-shaped pH profile through a lower specific activity in the acidic region than *Ba*L WT, while the specific activity at less acidic and neutral pH is not increased. pH-Type II defines variants showing a higher specific activity between pH 3.0 and 6.5 than *Ba*L WT, and pH-type III indicates higher specific activities above pH 6.5 than *Ba*L WT. Regardless of the type of effect attributed to the different amino acid exchanges, all variants except pH-type III variants show a considerable decrease in activity in the pH range of pH 6.0–7.0. This might be attributed to the protonation state of the T1 copper-coordinating histidine imidazole rings, which were previously reported to be protonated at pH 5.0^33^ and exhibit a pKa of ~6^34^, respectively. The role of the protonated T1-coordinating histidine as primary electron acceptor in catalysis was recently proposed^33,35,36^. Three mutations (D236E, I424G, L499F) resulted in increased specific activities above pH 6.5. I424G and L499F showed pH-type II + III with a 2.6- and 4.8-fold increased specific activity at pH 7.5, respectively. D236E showed pH-type II effects with ABTS (Fig. 2b), while all three variants exhibited pH-type II + III behaviour with DMP (Fig. 2a) with a 2.1-, 2.1-, and 2.0-fold increase of specific activity, respectively, at pH 7.5. Detailed datasets in the SI show both the relative and specific activities of the variants towards ABTS and DMP and include the corresponding -fold increases or decreases compared to *Ba*L WT covering the entire pH range (Supplemental Tables S7–S11).

### Kinetic characterisation of *Ba*L variants

**Figure 3:**
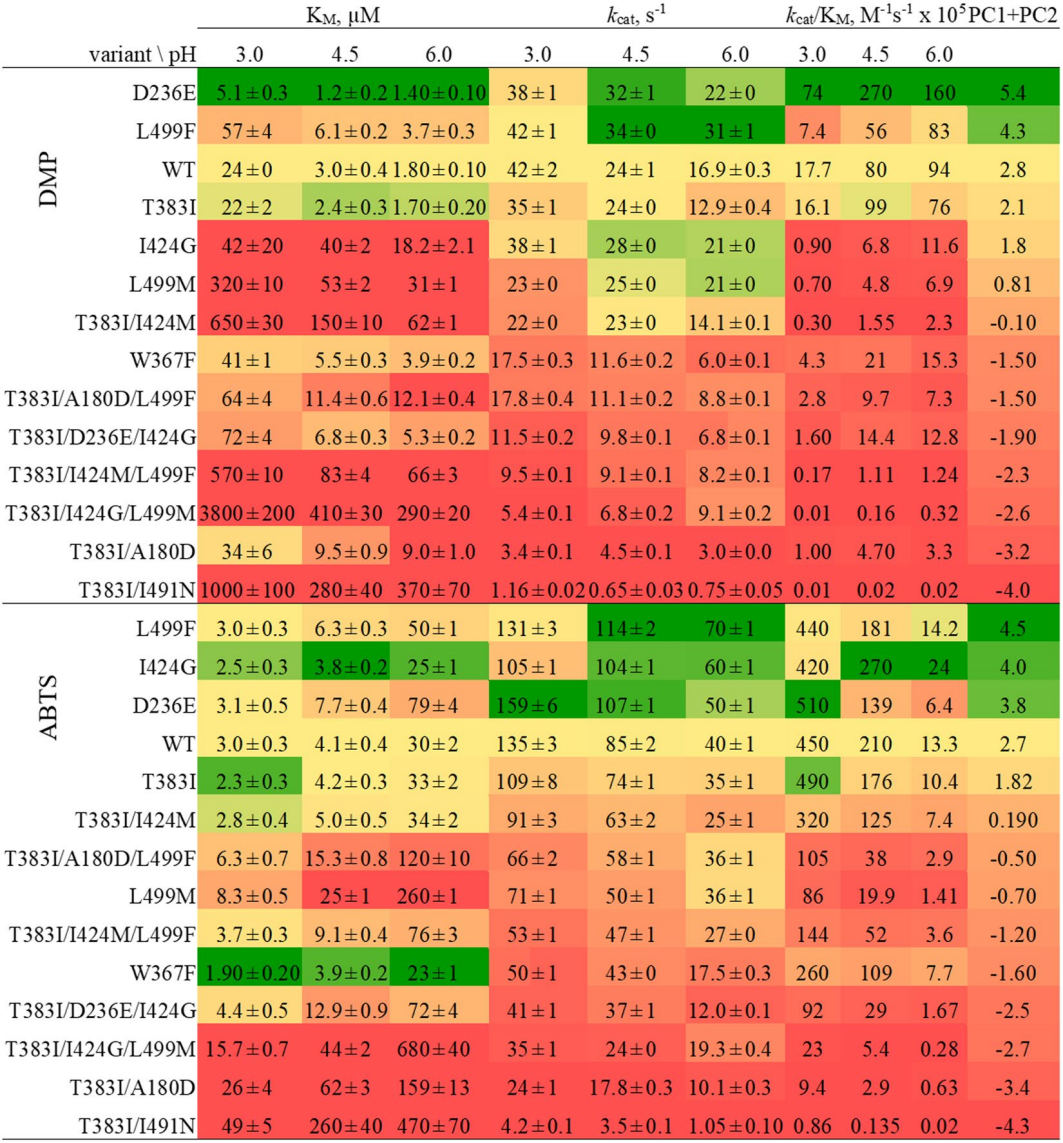
Apparent steady-state kinetic constants of *Ba*L variants for the substrates DMP and ABTS at pH 3.0, 4.5 and 6.0. Variants are arranged from top (best overall performer) to bottom (worst overall performer) by the sum of their principal components PC1 + PC2 (far right column) calculated by Principal Component Analysis (Fig. 5, Supplemental Tables S14 – S21). Colour code: yellow, WT levels; green: better than the WT; red: worse than the WT.

**Figure 4:**
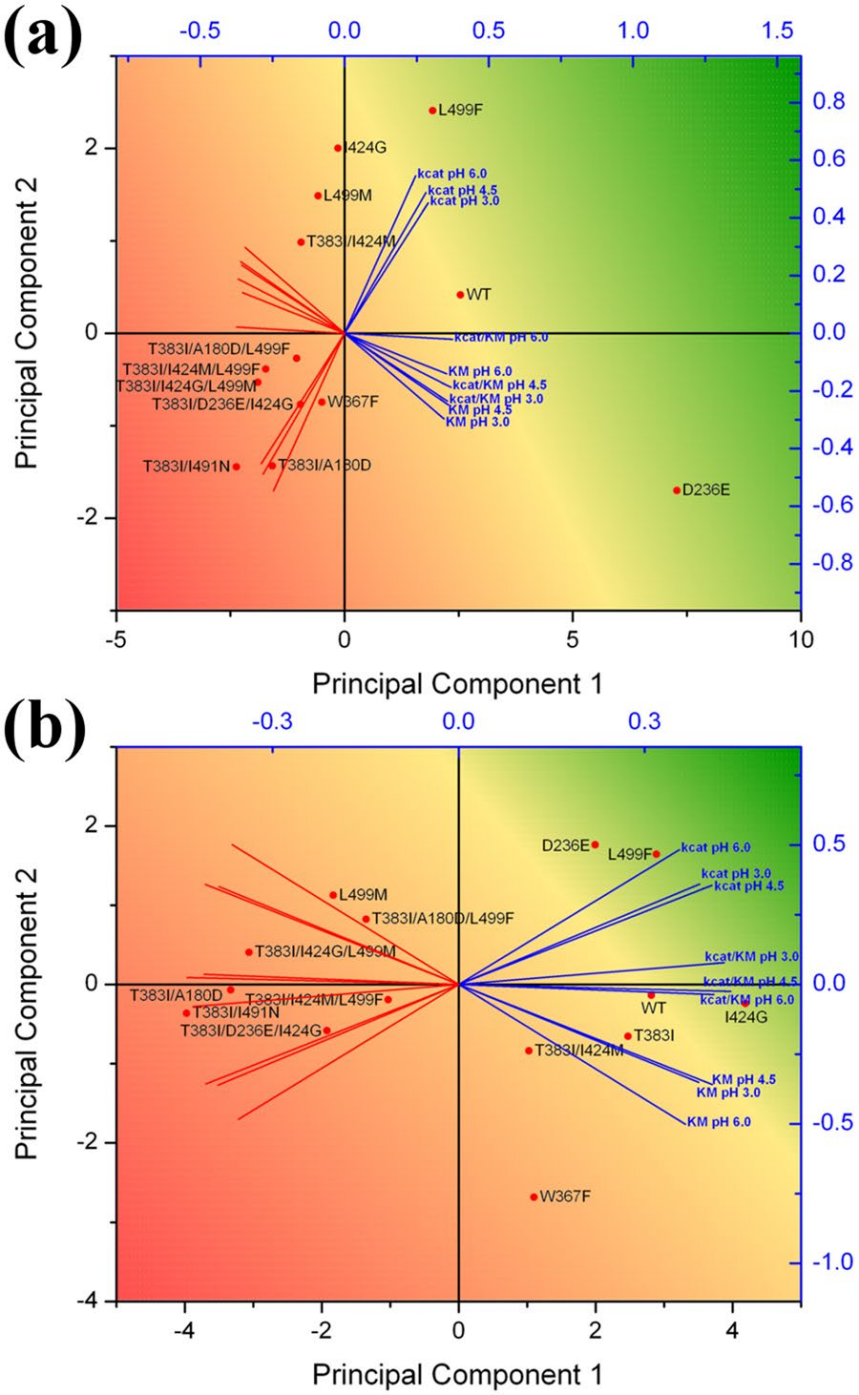
Principal Component Analysis (biplot) of the kinetic constants of 14 *Ba*L variants with (**a**) DMP and (**b**) ABTS. The *Ba*L variants are indicated by red dots (observations). Direction and length of the blue and red lines (variables) indicate the weight of positive and negative influence, respectively, of the respective kinetic constant, i.e.: L499F oxidizing DMP owes its positioning to improved catalytic constants especially at higher pH values while, for example, D236E oxidizing DMP also shows improved catalytic constants but owes its position to reduced K_M_ values especially at lower pH values. Colour code (corresponds to Fig. 3): yellow, WT levels; green: better than the WT; red: worse than the WT. Refer to Fig. 3 for the applied raw data.

The apparent steady-state kinetic constants were determined for the two substrates DMP and ABTS at three pH values (3.0, 4.5, and 6.0) for 14 *Ba*L variants as well as *Ba*L WT. These constants are shown in Fig.3 and graphically represented in Supplemental Fig. S3. The dataset of each substrate was subjected to a principal component analysis (PCA, Fig. 4, Supplemental Tables S14–S21) to identify the key determinants of the effect of the pH value on enzymatic activity. The biplot shows both loadings (weight of the variables) and scores (observations in the X/Y-space) for the two principal components and reveals the relations of variables (kinetic parameters at a specific pH) and observations (14 variants). It positions the variants with respect to their overall kinetic performance over the pH relative to each other. PCA shows that the catalytic efficiencies (*k*_cat_/K_M_) of *Ba*L variants for DMP increase with increasing pH whereas the catalytic efficiencies for ABTS decrease with increasing pH.

All amino acid exchanges, except D236E, increased the K_M_ for DMP, most notably I424G. In contrast, the K_M_ for ABTS typically increased with higher pH values, which resulted along with a decrease in *k*_cat_ in reduced catalytic efficiencies of *Ba*L variants at higher pH values. The effects of some mutations on *k*_cat_, for example L499F or I424G, were more pronounced at higher pH. A detailed analysis of changes in kinetic constants by pH of all 14 variants and relative to *Ba*L WT is given in Supplemental Tables S12 and S13.

### Effects of mutations

#### T383I

**Figure 5:**
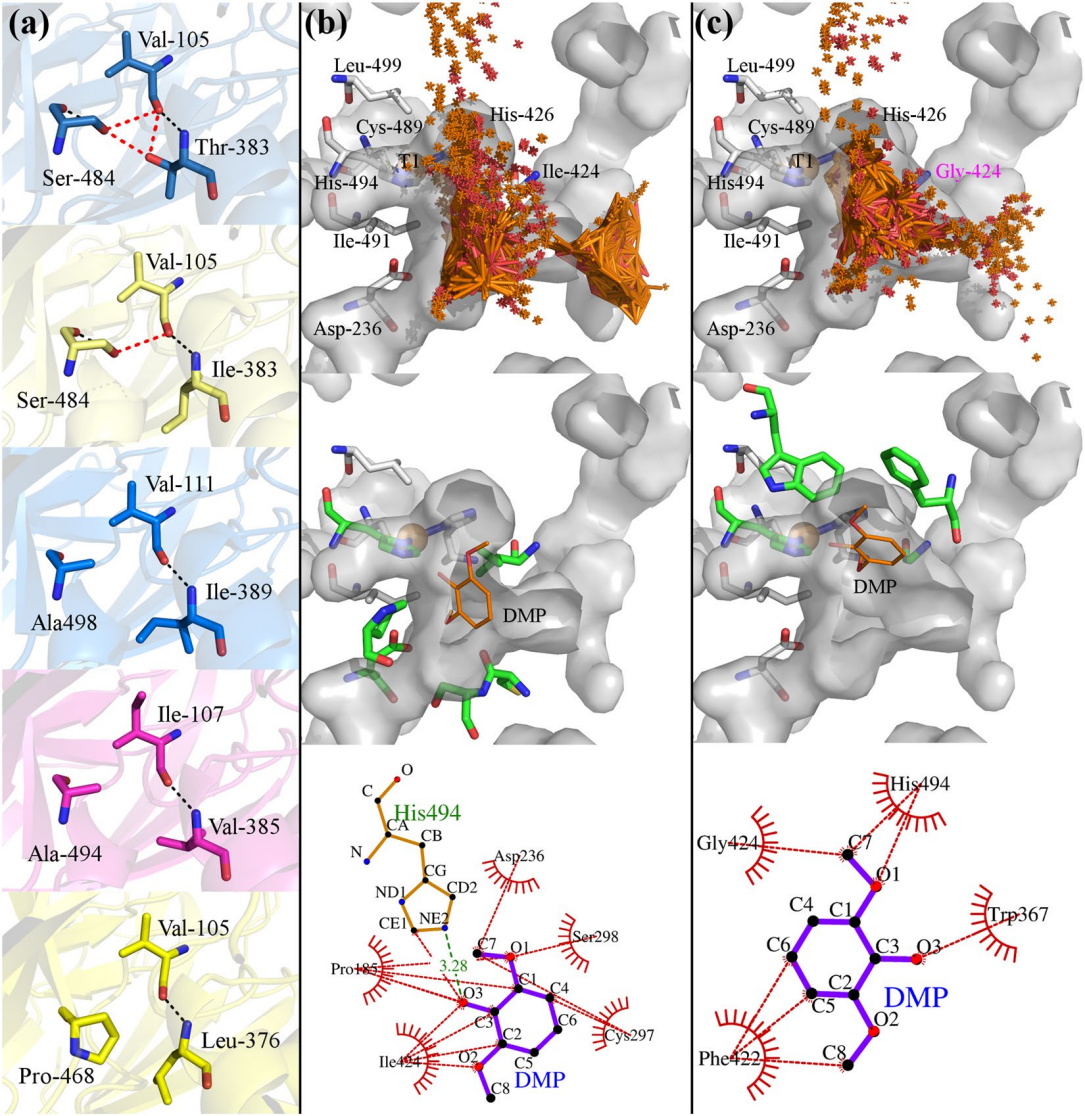
Structural environment of T383I (**a**, top to bottom) and dockings of DMP to *Ba*L WT (**b**) and *Ba*L I424G (**c**). Panel (**a**) shows from top to bottom the structural environment of T383 (top) and I383 in *Botrytis aclada* laccase and the corresponding positions in the thermostable laccases from *Melanocarpus albomyces* (PDB: 2Q9O, V111-I389-A498), *Myceliophthora thermophila* (Homology model; template, 1GW0, GMQE, 0.77, I107-V389-A494), and *Ceriporiopsis subvermispora* (Homology model; template, 3KW7; GMQE, 0.84, V105-L376-P468), respectively, and an overlay of interacting residues of thermostable laccases (bottom).  and  show conservative hydrogen bonds and destabilizing interactions, respectively. Table 2 provides data from thermostable laccases. Panel (b) and (c) show from top to bottom docking clusters of DMP (top) to *Ba*L WT (b) and *Ba*L I424G (c), the single DMP molecule closest to the catalytic histidine 494 (middle), and its interaction with the polypeptide (bottom), respectively. T1-coordinating- and axial ligands, D236 and I/G424 residues as well as the T1-copper are labelled. Green residues in the middle sections correspond to the DMP-interacting amino acids in the bottom sections, in which  and  indicate hydrogen bonds and hydrophobic interactions, respectively. The docking was done using the online tool SwissDock^45^. Ligand-protein interaction (panel c, bottom) was calculated with the software LigPlot^48^.

The above described results can be rationalised as follows: in addition to the α-helix _382–389_ stabilizing hydrogen bond between the T383 amine group and the V105 carbonyl group, three destabilizing interactions between the T383 β-hydroxyl and the V105 carbonyl, the T383 β-hydroxyl and the S484 β-hydroxyl, and the S484 β-hydroxyl and the V105 carbonyl groups are observed (Fig. 5a). The exchange of Thr by Ile retains the stabilizing H-bond while two out of three destabilizing interactions are removed, resulting in an increase of the solvation energy effect (ΔiG) from 0.26 (Thr) to 1.29 (Ile) kcal mol^−1^. (Supplemental Tables S3–S6). This is further supported by a comparison with other thermostable laccases (Table 2), all of which lack the destabilizing interactions caused by T383 in *Ba*L WT (Fig. 5a). The amino acid exchange T383I increases the thermodynamic stability of *Ba*L substantially, while it hardly affects its activity since the specific activity of this variant remains virtually unchanged with either DMP or ABTS as substrate compared to *Ba*L WT (Table 3).

**Table 2:** Comparison of thermostable fungal laccases.

	Organism	Enzyme	Res 1	Res 2	Res 3	T_opt_, [°C]	T_50_, [°C]	Substrate^a^	Reference
Ascomycetes	*Botrytis aclada*	WT	V	T	S	nr	69	ABTS	this study
	*Botrytis aclada*	T383I	V	I	S		75	ABTS	this study
	*Melanocarpus albomyces* (VTT D-96490)		V	I	A	60–70	60	ABTS, GUA	49,50
	*Myceliophthora thermophila* (CBS117.65)		I	V	A	nr	70	SGZ	51,52
Basidiomycetes	*Ceriporiopsis subvermispora*	L1	V	T	P	nr	60	ABTS	53
	*Pleurotus ostreatus*	POXC	V	L	P	50–60	60	ABTS	54
	*Pycnoporus cinnabarinus* (CBS 101046)		V	L	P	nr	80	SGZ	55
	*Pycnoporus sanguineus* (CCT-4518)		V	L	P	50	80	SGZ	56
	*Steccherinum ochraceum* (1833)	Laccase III	I	L	P	80	70	ABTS	57
	*Trametes sp*. (AH28–2)	LacA	V	L	P	50	75	GUA	58
	*Ganoderma lucidum* (KMK2)		I	L	P	60	60	ABTS	59
	*Basidiomycete* PM1 (CECT 2971)		V	L	P	80	nr	ABTS	60

Res 1, Res 2 and Res 3 indicate the amino acids found in these laccases in the corresponding positions of V105, T383 and S484 of *Botrytis aclada* laccase; T_opt_ indicates the optimal temperature reported for the laccase assay, T_50_ indicates the temperature at which 50% residual activity is retained.

^a^ABTS, 2,2′-azino-bis(3-ethylbenzothiazoline-6-sulfonic acid; GUA, guaiacol; SGZ, syringaldazine.

nr, not reported.

**Table 3:** Specific activities and improvement related to *Ba*L WT variants with DMP and ABTS as substrates.

substrate	variant	pH-type	pH opt.	specific activity, U mg^−1^						fold improvement		
				pH value						pH value		
				2.5		5.5		6.5		2.5	5.5	6.5
DMP	WT	—	2.5	49	±2	22	±1	14.5	±1.0	—	—	—
	D236E	II + III	2.5	36	±3	33	±1	23	±2	0.73	1.53	1.58
	L499F	II + III	3.0	34	±1	32	±1	21	±2	0.70	1.48	1.45
	I424G	II + III	3.5	25	±1	27	±0	16.4	±3.2	0.51	1.24	1.13
	T383I	—	2.5	40	±2	19.5	±0.5	12.6	±1.6	0.81	0.90	0.87
ABTS	WT	—	2.5	131	±3	56	±2	27	±1	—	—	—
	D236E	II	3.0	112	±4	83	±3	27	±1	0.85	1.48	1.00
	L499F	II + II	3.0	109	±8	89	±7	50	±7	0.83	1.59	1.85
	I424G	II + III	3.5	83	±6	67	±6	41	±5	0.64	1.20	1.52
	T383I	—	2.5	124	±6	51	±2	25	±2	0.95	0.91	0.92

Specific activities as well as the -fold improvements are given for pH 2.5, 5.5 and 6.5. -Fold improvements relate specific activities of the respective variants at a given pH to *Ba*L WT where values < 1 and > 1 mean decrease and improvement, respectively, compared to *Ba*L WT. For both substrates and all variants, the identified pH-type (see text for details) and the pH optima are given.

**L499** is the second axial, non-coordinating ligand of the T1 copper and is strongly conserved in ascomycetous laccases, whereas Phe is almost fully conserved in basidiomycetous laccases at this position. This copper ligand is opposite of the substrate-binding site, buried in the protein core, and is known to influence the redox potential of the T1 copper^37^.

The exchange **L499F** follows the prevalence of this amino acid in high redox potential basidiomycete laccases. This mutation increases *k*_cat_ for DMP and ABTS at pH 4.5 and 6.0, together with a slight increase of K_M_. Presumably, a change of the T1 redox potential is responsible for this change in kinetic constants and pH profile. L499F exhibits a somewhat lower activity in the acidic pH (2.5–4.0) compared to *Ba*L WT, but retains a continuously high specific activity throughout the whole pH range until pH 6.0 before starting to drop. L499F showed a broad activity maximum from pH 2.5 to pH 6.0 with DMP, retaining 82% of its maximum activity at pH 6.0. The specific activity between pH 4.0–8.0 is always higher than that of *Ba*L WT (pH-type II + III). This is also reflected by the *k*_cat_ values of L499F, which are similar to *Ba*L WT at pH 3.0 for both substrates, but increase with increasing pH (1.4- and 1.3-fold higher at pH 4.5 for DMP and ABTS, respectively, and 1.8-fold higher at pH 6.0 for both substrates).

**I424** is located in the substrate-binding site, 5–6 Å from the T1 copper. **I424G** changes size and shape of the substrate-binding site. This is reflected by changes in K_M_, whereas *k*_cat_ is only slightly affected. Investigation of the crystal structure and docking results reveal that I424 plays an important role in positioning of the phenolic substrate in the substrate binding pocket (Fig. 5b). I424G clearly enlarges the binding site (Fig. 5c) and the increased space together with the loss of the Ile side chain affects the exact placing of the phenolic substrate in the active site. Thus, the K_M_ for DMP is increased, whereas ABTS seems to be accommodated better by this larger binding site, as reflected by a decreased K_M_. Introducing Gly into this position in *Ba*L resulted in a reduction of specific activity at acidic pH (2.5–4.0) for both substrates ABTS and DMP but also in higher specific activities in the more alkaline region. I424G shifts the pH optimum from pH 2.5 to pH 3.5 for both substrates, reaching *Ba*L WT-levels at pH 4.5. With increasing pH, it loses less activity than *Ba*L WT (pH-type II + III). Compared to *Ba*L WT, variant I424G exhibits much lower catalytic efficiencies with DMP (19.7-fold, 11.8-fold and 8.1-fold at pH 3.0, 4.5 and 6.0, respectively) while converting ABTS as efficiently as *Ba*L WT at pH 3.0 and better at pH 4.5 (1.3-fold) and 6.0 (1.8-fold).

**D236** is also positioned in the substrate-binding site. The mutation D236E has been reported to deprotonate phenolic substrates thus increasing their turnover and changing the pH profile of laccases for phenolic substrates^13,24,36,38–40^. This is also true for *Ba*L D236E, where a decreased K_M_ and an increased *k*_cat_ for both DMP and ABTS was found. D236, located at the top of a loop about 7.5 Å apart from the T1 copper, is the only charged residue within the substrate-binding pocket of *Ba*L. In ascomyceteous *Thielavia arenaria* laccase it is the only hydrophilic residue among ten substrate pocket forming hydrophobic residues (40% sequence identity, 1.3 Å RMSD)^40^. The variant D236E showed a flattened plateau-like pH curve until reaching pH 6 for DMP and pH 5 for ABTS, leading to higher specific activities at pH values higher than 4.0 compared to *Ba*L WT. This exchange shifts the pH profile for both the phenolic and non-phenolic substrate DMP and ABTS, respectively, to the more alkaline pH range (pH-type II + III, Figs 2a,b and S3), while the observed effect is much more pronounced with DMP. Notably, we found that in site-saturation mutagenesis studies at position 236 only the exchange from aspartate to glutamate showed beneficial effects. It has been proposed that this residue is responsible for the different pH behaviour on phenolic substrates of plant (N) and fungal (D/E) laccases while action on ABTS was reported to be not affected because there is no proton exchange involved^28^. D236E exhibits maximum catalytic efficiency for DMP at pH 4.5 and converts the substrate 4.2-fold, 3.3-fold and 1.7-fold more efficiently than *Ba*L WT at pH 3.0, 4.5 and 6.0, respectively. With ABTS it is similar (pH 3.0) or less (1.5-fold at pH 4.5; 2-fold at pH 6.0) efficient than *Ba*L WT. Catalytic turnover numbers are slightly improved at elevated pH for both tested substrates.

## Conclusions

Shifting the pH profile of a HRPL towards neutral pH is a difficult endeavour, as can be seen in this as well as in previous studies^15,16^. Multiple mutations are necessary to achieve the desired activity^18,19^ but must be carefully balanced in order to neither jeopardise the redox potential nor the kinetic properties of the enzyme. A fine-tuning of pH-dependent activity and pH-dependent redox potentials of both copper sites is necessary, otherwise the electron transfer between the centres is compromised. By applying various protein engineering and evolution approaches three *Ba*L variants with up to 5-fold increased specific activity at pH 7.5 and one *Ba*L variant with increased thermostability were obtained. Compared to published data, the here reported specific activities of up to 13.4 U mg^−1^ (L499F, ABTS) at pH 7.5 are unreached so far among fungal HRPLs. Together with an increase of thermal stability (T_50_) of 7 °C, this result is of great importance for the application in implantable enzymatic biocathodes. The obtained pH profiles showed that I424G admittedly reduces specific activity in the far acidic pH but exceeds *Ba*L WT activities with increasing pH. D236E and L499F, in contrast, shift the pH profile to the less acidic region with both ABTS and DMP. Our data from the random directed evolution approach show that most of the amino acid exchanges found by applying selective pressure via an elevated pH value cluster around or are close to the T1 copper centre. We therefore suggest, that in addition to the previously suggested inhibition of the trinuclear copper cluster by hydroxide anions^41^, also the T1 copper centre as well as second-shell amino acids, as shown by various mutational studies^14,15,19,42^, modulate enzymatic activity and electron transfer in respect to pH and restrict the activity of HRPLs to acidic pH. To further elucidate the role of pH-dependent ligand protonation and OH^−^ inhibition on the T1 copper centre, inhibition studies in combination with the determination of T1 redox potentials by electrochemical and EPR studies will be of great value but exceed the scope of this study.

## Materials and Methods

### Chemicals

All chemicals used were of analytical grade or highest purity available. All buffers and aqueous solutions were prepared with deionised water (>17 MΩ). 2,2′-azino-bis(3-ethylbenzothiazoline-6-sulphonic acid) (ABTS) and yeast nitrogen base were from Amresco LLC (OH, USA). 2,6-dimethoxyphenol (DMP), acetic acid, boric acid, magnesium chloride, manganese sulphate, peptone from casein, potassium hydroxide, potassium sulphate, sodium hydrogen phosphate, sodium molybdate and sulfuric acid were from Fluka (St. Gallen, CH). Zeocin was from Invitrogen (Carlsbad, CA, USA). Sodium hydroxide was from Merck (Darmstadt, Germany). Agar, ammonium sulphate, glucose, glycerol, methanol, phosphoric acid (85%), potassium sulphate, potassium dihydrogen phosphate, potassium hydrogen phosphate and sodium chloride were from Roth (Karlsruhe, Germany). Agarose, biotin, calcium sulphate, citric acid, cobalt chloride, copper sulphate, EDTA, ferrous sulphate, magnesium sulphate, sodium iodide, Tris base, yeast extract and zinc chloride were from Sigma Aldrich (St. Louis, USA). Ethanol was from VWR (Radnor, USA). *Escherichia coli* NEB5α (New England Biolabs, Ipswich, MA, USA) was used for subcloning and *Pichia pastoris* × 33 (Invitrogen) for heterologous expression^9^. A modified pGAPZ A vector (Invitrogen) under the control of the GAP promotor was used for expression in *P*. *pastoris* ^43^.

### Enzyme Production and Purification

*Botrytis aclada* laccase wild-type and variants were recombinantly produced in *P*. *pastoris* and purified as described by Kittl *et al*.^9,43^. Purification was carried out pursuing a three-step strategy comprising initial cell removal by centrifugation (6000 x *g*, 4 °C, 30 min) using an Optima L-70 Ultracentrifuge (Beckman Coulter, Pasadena, CA, USA), a primary precipitation step with 40% (NH_4_)_2_SO_4_, and two consecutive hydrophobic interaction chromatography steps on Phenyl Sepharose and Phenyl SOURCE^TM^ columns.

### Creation of mutagenic libraries

Both mutagenic libraries were created by error-prone PCR (Supplemental Tables S22–S28) with mutational rates of 2.1 and 1.8 mutations per 1000 base pairs, respectively, in a C1000 Thermal Cycler (BioRad, Hercules, CA, USA) using a modified pPICZA plasmid^43^ containing the *Ba*L cDNA. The amplified PCR products were separated from the residual template plasmid DNA by agarose gel electrophoreses, purified, digested with *Not*I and *Nde*I (37 °C, overnight) and purified again. Similarly, a plasmid backbone for ligation was created by digesting the pGAPZ A vector with *Nde*I and *Not*I (37 °C, overnight). The previously purified insert was then ligated with T4 DNA ligase into the backbone DNA by applying an optimised ligation temperature program (16 steps, 60 min/step, +0.5 °C/step, 10 °C starting temperature, final hold at 4 °C). The ligation mixtures were transformed into chemically competent *E*. *coli* NEB5α for proliferation. Plasmids were isolated (PureYield Plasmid Miniprep System, Promega, Madison, WI, USA), linearised with *Pag*I and transformed into electrocompetent *P*. *pastoris* x 33 cells for expression.

Site-saturation and site-directed mutagenesis were used to create a series of enzyme variants and were performed as described elsewhere^7,9,43^. Briefly, a PCR with the corresponding primer pairs (Supplemental Tables S29 and S30) and the expression vector p*Ba*Lac^43^ based on pGAPZ A (Invitrogen) containing the cDNA of *Ba*L was performed. PCR products were digested with *Dpn*I for 1 h at 37 °C to remove residual template plasmids and transformed into chemically competent *E*. *coli* NEB5α cells.

Alternatively, two halves of the insert were amplified separately with a vector-specific forward primer (pGAPfw2), a point-mutation-carrying reverse primer, an overlapping point-mutation-carrying forward primer and a vector-specific reverse primer (3ZLKGAP1), respectively. In a second PCR, the resulting PCR products were fused to a single point mutation-carrying fragment with the primers pGAPfw2 and 3ZLKGAP1. The fusion PCR product was digested with *Nde*I and *Not*I, ligated into equally digested pGAPZ A with T4 DNA-ligase (1 h, room temperature) and transformed into chemically competent *E*. *coli* NEB5α cells.

Transformants were selected using Zeocin as selection marker and plasmids were isolated using the PureYield Plasmid Miniprep Kit. Mutations were confirmed by sequencing (Microsynth, Balgach, CH) and plasmids were transformed into electrocompetent *P*. *pastoris* × 33.

Combinatorial mutagenesis was done as described above. In total, 24 combinations of eight point mutations having T383I as parent were created. A scheme showing a list of all variants created is shown in Fig. 1.

### Pre-screening

Positive *Pichia* transformants were selected on YPD-agar plates containing Zeocin (100 µg mL^−1^) as selection marker and replicated by a stamping technique using a sterile velvet cloth to three BMG-agar plates (pH 5.0, 6.0 and 7.5) containing 2 mM ABTS. Colonies showing active laccase expression (green halos caused by ABTS oxidation) at pH 6.0 after 12 h incubation at 30 °C were selected for the high-throughput screening, transferred with a sterile toothpick to a 96-well plate containing BYPD, pH 5.0, and incubated at 25 °C and 380 rpm in an Infors HT incubation shaker (Infors, Bottmingen, CH). Media abbreviations and composition are listed in Supplemental Tables S31 and S32.

### High-throughput differential (HT) screening

After incubation, *P*. *pastoris* cells were precipitated by centrifugation (3000 rpm, 10 min) using an Eppendorf Centrifuge 5804 (Eppendorf, Hamburg, DE). The clear supernatant was quadruplicated with the help of a JANUS® Automated Workstation (PerkinElmer, Waltham, MA, USA) for employment of a differential screening that allows evaluation of thermal stability and elevated activity at a pH in the neutral range. The activity of *Ba*L clones using ABTS and DMP as substrates was determined under three conditions – (1) pH 5.0 (reference), (2) pH 5.0 after incubation at 55 °C for 30 min, and (3) pH 6.5. The screening strategy is shown schematically in Supplemental Fig. S1. Identification of beneficial clones was done by first relating activities at testing conditions to activities at pH 5.0 and then relating the obtained relative activities to the relative activities of the respective parental enzyme. Final selection criteria for clones revealing beneficial effects were the R^2^ of the kinetic slope obtained from the activity assays and the standard deviation of the activities determined for the respective parental enzyme measured in quintuplicates. Clones with an R^2^ greater than 0.7 and a relative activity higher than the threefold standard deviation of the parental enzyme kinetic slope were rescreened to confirm the supposed beneficial effects, sequenced to identify the mutations, and subjected to recombinant protein production.

### Activity measurements

Laccase volumetric activity (U mL^−1^) was determined in 96-well microplates by following the oxidation of 1 mM ABTS at 420 nm (ε_420_ = 36000 M^−1^ cm^−1^) and 1 mM DMP (ε_469_ = 49600 M^−1^ cm^−1^), respectively, for 180 s in air-saturated 0.1 M citrate-phosphate buffer, pH 4.0. The absorbance was recorded with a 2300 EnSpire Multilabel Platereader (Perkin Elmer, Waltham, MA, US). Specific activities (U mg^−1^) were calculated as the ratio of volumetric activity and protein concentration.

### Thermostability

Thermostability was examined by means of time- (t) and temperature- (T) dependent residual activity. Laccase was incubated for 0, 1, 5, 10, 15, 30, 45 and 60 minutes at 55 °C for assessing the time-dependent and at 22, 37, 40, 45, 50, 55, 60 and 65 °C for 10 min for determining the temperature-dependent thermal stability. The time and the temperature at which 50% residual activity were retained were defined as t_50_ (half-life time) and T_50_ (temperature of half-inactivation), respectively. The laccase solution was cooled on ice for 60 s before being subjected to the laccase standard activity assay.

The transition midpoint temperature was measured by differential scanning calorimetry (DSC) using a MicroCal VP-DSC instrument with an autosampler (MicroCal, Northampton, MA). Laccase concentrations were adjusted to 1.0 mg mL^−1^ based on their molar extinction coefficients at 280 nm. All measurements were carried out in 100 mM citrate-phosphate buffer, pH 4.5. A linear temperature gradient from 50 °C to 90 °C was applied at a scan rate of 1 K min^−1^. Obtained thermograms were baseline-corrected by subtracting the buffer blank. Transition midpoint temperatures (T_m_) of the enzymes were determined from the peak maximum of the transition peak using the Origin 9.1 software (Origin Lab Corporation, Northhampton, MA, US)^44^.

Using the online tool PDBePISA (http://www.ebi.ac.uk/pdbe/pisa/), structural and thermodynamic parameters of the interface between a motif comprising 31 residues (I369-V399) hosting T383 or the modelled I383, respectively, and the entire enzyme without the specified motif were calculated.

### Protein characterisation

Purified *Ba*L variants were characterised using steady-state photometric methods to determine K_M_ and v_max_ by non-linear curve-fitting to the Michaelis-Menten equation using a least square minimization method. The molecular mass (62628.6 Da) was calculated from the amino acid sequence with the online tool ProtParam (http://web.expasy.org/protparam/). *k*_cat_ was derived from v_max_, the molecular mass and the enzyme concentration.

pH profiles for ABTS and DMP were recorded by applying the laccase standard activity assay and adjusting the pH from 2.5 to 8.0 in 0.5 pH-unit steps. Relative pH profiles relate the measured activity of individual laccase variants at the respective pH to the activity at their particular pH-optimum.

### Principal Component Analysis

The kinetic data of 14 *Ba*L variants comprising K_M_, *k*_cat_, and *k*_cat_/K_M_ values at pH 3.0, 4.5 and 6.0, respectively (Fig. 3), were analysed by principal component analysis for the substrates DMP and ABTS using the software OriginPro 8.6 (OriginLab). For both substrates, a correlation matrix was analysed and the number of components to extract was set to 2. The kinetic parameters at a specific pH serve as variables and the 14 variants as observations. The sum of the extracted principal components 1 and 2 was used as indicator for the overall performance of the respective variant.

### Dockings

Dockings of 2,6-dimethoxyphenol (DMP, syringol) to *Botrytis aclada* WT and I424G were done using the online tool SwissDock (www.swissdock.ch,^45^) applying the default settings. The region of interest was defined by the center of the coordinates of the α-carbons of A184, S298 and I424 and was extended allowed to extend by maximal 10 Å. The crystal structure of *Ba*L WT (PDB: 3SQR) and I424G (mutation introduced via homology modelling, see Supplemental Information) were prepared using the DockPrep plugin for Chimera^46^. The structure of syringol was downloaded from the ZINC database^47^.

### Protein-Ligand Interaction

After inspection of the docking results, the DMP molecule with least distance to the catalytic histidine was selected and together with the laccase structure used for protein-ligand interaction calculations. The calculations were done using the software LigPlot^48^.

### Data Availability

All data generated and/or analysed during this study are included in this published article (and its Supplemental Information files) or are available from the corresponding author on reasonable request.

## Electronic supplementary material

Supplemental Information
