# Supplementary material for: Evolving stability and pH-dependent activity of the high redox potential Botrytis aclada laccase for enzymatic fuel cells
Source: Sci Rep. 2017 Oct 20;7:13688. doi: 10.1038/s41598-017-13734-0 (PMC5651918; doi:10.1038/s41598-017-13734-0)
Supplement: Supplementary file 1 — Supplemental Information [file 41598_2017_13734_MOESM1_ESM.pdf]

Supplemental Information for

Evolving stability and pH-dependent activity of the high redox potential *Botrytis aclada* laccase for enzymatic fuel cells

Stefan Scheiblbrandner[1], Erik Breslmayr[1], Florian Csarman[1], Regina Paukner[1], Johannes Führer[1], Peter L. Herzog[1], Sergey V. Shleev[2], Evgeny M. Osipov[3], Tamara V. Tikhonova[3], Vladimir O. Popov[3], Dietmar Haltrich[1], Roland Ludwig[1], Roman Kittl[1*]

[1] Department of Food Sciences and Technology, VIBT – Vienna Institute of BioTechnology, BOKU–University of Natural Resources and Life Sciences, A-1190 Vienna, Austria

[2] Biomedical Sciences, Health and Society, Malmö University, 20560 Malmö, Sweden

[3] Russian Academy of Sciences, A.N. Bach Institute of Biochemistry, 119071 Moscow, Russian Federation

*Correspondence and requests for materials should be addressed to R.K (Phone: +43 1 47654-75215; Fax: +431 47654 75039; E-mail: roman.kittl@boku.ac.at)

this file contains:

Supplemental Text

Supplemental Tables S1 – S32

Supplemental Figures S1 – S4

Supplemental References

Erik Breslmayr: erik.breslmayr@boku.ac.at

Florian Csarman florian.csarman@gmx.net

Johannes Führer johannes.fuehrer@boku.ac.at

Dietmar Haltrich dietmar.haltrich@boku.ac.at

Peter L. Herzog peter.herzog@boku.ac.at

Roman Kittl roman.kittl@boku.ac.at

Roland Ludwig roland.ludwig@boku.ac.at

Evgeny M. Osipov e.m.osipov@gmail.com

Regina Paukner reginapaukner@gmx.at

Vladimir O. Popov vpopov@exchange.inbi.ras.ru

Stefan Scheiblbrandner: stefan.scheiblbrandner@boku.ac.at

Sergey V. Shleev sergey.shleev@mah.se

Tamara V. Tikhonova ttikhonova@inbi.ras.ru

Supplemental Text

Results & Discussion

Increased thermostability

T383 is one of eight amino acids that make up an alpha – helix located in the protein surface rather far away from both the T1 – copper and the T2/T3 – copper cluster. The side chain is facing inside the protein. The electron density of the crystal structure 3SQR shows that the threonine has two potential conformations, indicating a high flexibility of the region. This is supported by the higher B-factor of the crystal structure in this area indicating a higher mobility of the helix from N363 to N388. T383 forms hydrogen bonds to V105 and S484 (Fig. 2) and neighbours two N-glycosylation sites (N370 and N389).

Upstream of V105 the T2 copper coordinating H87 and T3b copper coordinating H89 residues are situated. S484 is located in a beta-strand, which is, in turn, part of a beta-sheet flanking the trinuclear copper cluster. The beta-sheet together with a beta hairpin hosts the T3a coordinating H431 and H488, the T2 coordinating H429, the T3b coordinating H490 and the T1 coordinating C489 and H494. N370 is located at the end of a beta-strand comprising a two-stranded sheet connected by solvent exposed hairpin with a high B-factor, suggesting high flexibility as well. N389 is located in a solvent exposed loop with a high B-factor in the crystal structure, indicating elevated flexibility, adjacent to the helix hosting T383.

A multiple sequence and structure alignment of 63 fungal, plant and bacterial laccases (Supplemental Fig. S4) showed that ascomycetes rather have a threonine, isoleucine or leucine and basidiomycetes have a conserved leucine at the position of *Ba*Ls T383. Notably, the laccase closest related to *Ba*L, the laccase from *Melanocarpus albomyces*, has an isoleucine at the corresponding position. Both basidiomycetes and ascomycetes prefer a hydrophobic residue (Val or Ile) at the corresponding positions to V105. Plant laccases have a conserved isoleucine here. S484 is rather a Ser or an Ala in ascomycetes, a highly conserved proline in basidiomycetes and a highly conserved valine in plant laccases. N370 is quite conserved among ascomyceteous, basidiomyceteous, plant and bacterial laccases whereas N389 is often an asparagine or glycine in ascomyceteous, and a conserved alanine in basidiomyceteous laccases.

A similar alignment of reported thermotolerant and thermostable laccases [1] revealed that a leucine is clearly preferred at T383 corresponding positions (Table 2). The only exceptions (besides *Ba*L) are laccases from *Melanocarpus albomyces* (I389; Fig. 2), *Myceliophthora thermophila* (V385; Fig. 2) *Ceriporiopsis rivulosa* (*Physisporinus rivulosus)* (T377; Fig. 2).

The change from a polar threonine to a hydrophobic isoleucine might stabilize the hydrophobic core of the protein. Furthermore, in a modelled (SwissModel) structure I383 only binds to V105 but not to S484 (Fig. 2). The freedom in movement of the loop N388 – P394 hosting the N-glycosylation site N389 and the beta-hairpin – beta-sheet F361 – S371 hosting the N-glycosylation site N370 might thus be supported.

Shifting laccase activity towards neutral pH

With DMP all variants have a pH-profile deviating from the wild-type while with ABTS as substrate effects were only minor (Supplemental Fig. S2).

With DMP the variants W367F, T383I/A180D, T383I/D236E/I424G, L499M and T383I/A180D/L499F showed an exclusive pH-type II behaviour with increasing intensity of the observed effect, while D236E and L499F exhibit a mixture of pH-type II+III (Supplemental Fig. S2a, first six panels from upper left to lower right). The activity of the variants D236E and L499F stayed almost constant between pH 3.0 and pH 5.5 and remained higher at neutral pH.

With ABTS the variants W367F and T383I/A180D and T383I/I491N showed no effects while the variants D236E, T383I/I424M, D236E, T383I/D236E/I424G, T383I/A180D/L499F, T383I/I424G/L499M, and T383I/I424M/L499F showed pH-type II effects and L499F pH-type II+III. pH-type II effects were most pronounced in D236E and L499F showing an only slowly decreasing plateau from pH 2.5 to pH 4.5 and pH 5.5, respectively, similar to the behaviour observed with DMP but less distinct.

L499F additionally retained more activity with further increasing pH than the wild-type and was the only variant having an increased activity at neutral pH with ABTS. The double variant combining A180D and L499F adopted L499Fs pH-profile for both substrates, although showing slightly decreased activity at higher pH, attributable to A180D, compared to the L499F single variant. In the double variant hosting both D236E and I424G, D236E seems to be responsible for abrogating the loss of activity with DMP at pH 2.5 and 3.0 of the single variant I424G, while

I424G might account for tempering the stable plateau from pH 3.0 to 5.5 of the D236E single variant. Effects with ABTS are similar.

The variants I424G and T383I/I491N showed with DMP a mixture of pH-type I+II+III with a pH optimum at 3.5, 4.5 and 4.0, respectively. Both variants exhibited a 3-phasic pH-profile establishing a peak in the rather acidic range and a decreasing plateau in the range from pH ~3.5 to 6.0 – 6.5 before losing activity after pH 6.5 but retaining higher relative activity in the neutral pH range. With ABTS L499M is the only variant that is type I+II with a pH optimum shift to pH 4.0.

I424G showed similar to D236E and L499F but less pronounced pH-type II effects with ABTS exhibiting a slight shift of the pH optimum and thus could be considered partly pH-type I.

T383I/I424G/L499M is the only variant showing exclusive pH-type I effects with DMP, while T383I/I424M and T383I/I424M/L499F are pH-type I+III. They exhibit pH optima of 5.0, 5.0 and 5.5, respectively.

The combination of I424M and L499F results in a more pronounced bell-shaped curve than found in I424M alone with DMP while with ABTS I424M only tunes down the effect of L499F. The combination of the two mutations L499M and I424G results in the most pronounced pH-type I bell-shaped pH-profile.

A summary of all found effects including major data is given in Table S11.

Kinetic characterisation of *Ba*L variants

A set of all tested variants showing kinetic data assorted by catalytic efficiency at pH 6.0 from highest to lowest is illustrated in Supplemental Fig. S3. The kinetic behaviour of each variant relative to pH 3.0 and relative to *Ba*L wild-type is described in detail for both tested substrates.

DMP

In the following, the behaviour of catalytic constants in dependency of increasing pH for the substrate DMP is described for each variant. Percentages of retention or factors of improvement are always related to values at pH 3.0 set to 100 % or 1, respectively, and are always stated first for pH 4.5 followed by pH 6.0. Example: *Increase of substrate affinity from pH 3.0 to 4.5 and 6.0*

by a factor of x and y, respectively. To reduce the amount of redundancy, the term "*from pH 3.0 to 4.5 and 6.0 (...), respectively.*" was omitted in the following.

WT. Increase of substrate affinity by a factor of 7.9 and 13.1. Decrease of catalytic turnover retaining 58 and 40 %. Increase of catalytic efficiency by a factor of 4.5 and 5.3.

T383I/I424G/L499M. Moderate to strong increase of substrate affinity by a factor of 9.3 and 13.3. Increase of catalytic turnover by a factor of 1.26 and 1.69, that is, it is the only variant showing increasing turnover numbers with increasing pH. Strong increase of catalytic efficiency by a factor of 11.7 and 22.7.

T383I/A180D. Weak increase of substrate affinity by a factor of 3.6 and 3.8. Increase of catalytic turnover by a factor of 1.31 from pH 3.0 to 4.5 and decrease retaining 89 % from pH 3.0 to 6.0, that is, it shows highest turnover numbers at pH 4.5 instead of 3.0. Moderate to weak increase of catalytic efficiency by a factor of 4.7 and 3.4, that is, it shows highest efficiency at pH 4.5 instead of 6.0.

L499M. Moderate to weak increase of substrate affinity by a factor of 6.1 and 10.3. Weak decrease of catalytic turnover retaining ~100 and 93 %. Strong increase of catalytic efficiency by a factor of 6.8 and 9.8.

T383I/I424M/L499F. Moderate to weak increase of substrate affinity by a factor of 6.9 and 8.5. Weak decrease of catalytic turnover retaining 96 and 87 %. Strong increase of catalytic efficiency by a factor of 6.5 and 7.3.

T383I/D236E/I424G. Moderate to strong increase of substrate affinity by a factor of 10.6 and 13.6. Weak decrease of catalytic turnover retaining 85 and 59 %. Strong increase of catalytic efficiency by a factor of 9.0 and 8.0, that is, it shows highest efficiency at pH 4.5 instead of 6.0.

L499F. Strong increase of substrate affinity by a factor of 9.3 and 15.3. Weak decrease of catalytic turnover retaining 81 and 73 %. Strong increase of catalytic efficiency by a factor of 7.6 and 11.3.

T383I/I424M. Weak increase of substrate affinity by factor of 4.3 and 10.5. Weak to moderate decrease of catalytic turnover retaining ~100 and 63 %. Moderate increase of catalytic efficiency by a factor of 5.2 and 7.6.

D236E. Weak increase of substrate affinity by factor of 4.3 and 3.6, that is, it shows highest substrate affinity at pH 4.5 instead of 6.0. Weak decrease of catalytic turnover retaining 85 and 58

%. Weak increase of catalytic efficiency by factor of 3.5 and 2.6, that is, it shows highest catalytic efficiency at pH 4.5 instead of 6.0.

I424G. Strongest increase of substrate affinity by a factor of 10.5 and 23.3. Weak to moderate decrease of catalytic turnover retaining 72 and 55 %. Strong increase of catalytic efficiency by a factor of 7.6 and 12.9.

W367F. Moderate to weak increase of substrate affinity by a factor of 7.4 and 10.4. Moderate to strong decrease of catalytic turnover retaining 66 and 34 %. Weak increase of catalytic efficiency by a factor of 4.9 and 3.6, that is, it shows highest efficiency at pH 4.5 instead of 6.0.

T383I/A180D/L499F. Weak increase of substrate affinity by a factor of 5.6 and 5.3. Moderate to strong decrease of catalytic turnover retaining 62 and 50 %. Weak increase of catalytic efficiency by a factor of 3.5 and 2.6, that is, it shows highest efficiency at pH 4.5 instead of 6.0.

T383I/I491N. Weak increase of substrate affinity by a factor of 3.6 and 2.7. Strong decrease of catalytic turnover retaining 56 and 64 %. Weak increase of catalytic turnover by a factor of 2.0 and 1.8. that is, it shows highest efficiency at pH 4.5 instead of 6.0.

In the following, the absolute behaviour of kinetic constants relative to the WT for the substrate DMP is described for each variant. Percentages of yields are always related to values of the WT set to 100 % and are always stated for pH 3.0 first, followed by pH 4.5 and 6.0. Example: *High substrate affinities yielding x, y and z % of the wild-type at pH 3.0, 4.5 and 6.0, respectively.* To reduce the amount of redundancy, the term "*of the wild-type at pH 3.0, 4.5 and 6.0, respectively.*" was omitted in the following.

D236E. High substrate affinities yielding 462, 250 and 129 %, that is, it shows highest affinities over the pH of all tested variants. High catalytic turnover yielding 91, 134 and 131 %, that is, it shows the third, second and second highest turnover numbers at pH 3.0, 4.5 and 6.0, respectively, of all tested variants. High catalytic efficiency yielding 419, 334 and 169 %, that is, it shows the highest efficiency over the pH of all tested variants.

L499F. Moderate substrate affinity yielding 42, 49 and 49 %. High catalytic turnover yielding 101, 142 and 183 %, that is, it shows the highest catalytic turnover over the pH of all tested variants.

Moderate to high catalytic efficiency yielding 42, 70 and 89 %, that is, it shows the second highest efficiency at pH 6.0 of all tested variants.

I424G. Low substrate affinity yielding 5.6, 7.4 and 9.9 %. High catalytic turnover yielding 91, 115 and 125 %, that is, it shows the second and third highest catalytic turnover at pH 3.0 and 4.5, respectively. Low catalytic efficiency yielding 5.1, 8.5 and 12.4 %.

L499M. Low substrate affinity yielding 7.3, 5.7 and 5.8 %. Moderate to high catalytic turnover yielding 55, 104, and 127 %. Low catalytic efficiency yielding 4.0, 5.9 and 7.3 %.

T383I/I424M. Low substrate affinity yielding 3.6, 2.0 and 2.9 %, that is, it shows the third lowest substrate affinity at pH 3.0 and 4.5 of all tested variants. Moderate to high catalytic turnover yielding 53, 97 and 84 %. Low catalytic efficiency yielding 1.7, 1.9 and 2.4 %.

W367F. Moderate substrate affinity yielding 58, 55, and 46 %. Moderate to low catalytic turnover yielding 42, 48 and 35 %, that is, it shows the third lowest turnover numbers at pH 6.0 of all tested variants. Moderate catalytic efficiency yielding 24, 26 and 16 %.

T383I/A180D/L499F. Moderate to low substrate affinity yielding 37, 26 and 15 %. Moderate to low catalytic turnover yielding 43, 46 and 52 %. Moderate to low catalytic efficiency yielding 2+, 12 and 7.8 %.

T383I/D236E/I424G. Moderate substrate affinity yielding 33, 44 and 34 %. Moderate to low catalytic turnover yielding 28, 41 and 40 %. Moderate catalytic efficiency yielding 9.0, 18 and 14 %.

T383I/I424M/L499F. Low substrate affinity yielding 5.3, 3.6 and 2.7 %, that is, it shows the third lowest substrate affinity at pH 6.0 of all tested variants. Low to moderate catalytic turnover yielding 23, 38, and 49 %. Low catalytic efficiency yielding 0.9, 1.4 and 1.3 %, that is it shows the third lowest efficiency at pH 3.0, 4.5 and 6.0 of all test variants.

T383I/I424G/L499M. Low substrate affinity yielding 0.6, 0.7 and 0.6 %, that is, it shows the lowest at pH 3.0 and 4.5 and second lowest at pH 6.0 substrate affinities of all tested variants. Low to moderate catalytic turnover yielding 13, 28 and 54 %, that is, it shows the third lowest turnover numbers at pH 3.0 and 4.5 of all tested variants. Low catalytic efficiency yielding 0.08, 0.2 and 0.3 %, that is, it shows the second lowest efficiency at pH 3.0, 4.5 and 6.0 of all tested variants.

T383I/A180D. Moderate substrate affinity yielding 69, 31 and 20 %. Low catalytic turnover yielding 8.2, 19 and 18 %, that is, it shows the second lowest turnover numbers at pH 3.0, 4.5 and 6.0 of all tested variants. Low catalytic efficiency yielding 5.6, 5.8 and 3.6 %.

T383I/I491N. Low substrate affinity yielding 2.3, 1.1 and 0.5 %, that is, it shows the second lowest at pH 3.0 and 4.5 and lowest at pH 6.0 substrate affinity of all tested variants. Low substrate affinity yielding 2.8, 2.7 and 4.5 %, that is, it shows the lowest turnover numbers at pH 3.0, 4.5 and 6.0 of all tested variants. Low catalytic efficiency yielding 0.06, 0.03 and 0.02 %, that is, it shows the lowest efficiencies at pH 3.0, 4.5 and 6.0 of all tested variants.

ABTS

In the following, the behaviour of kinetic constants in dependency of increasing pH for the substrate ABTS is described for each variant. Percentages of retention always related to values at pH 3.0 set to 100 % and are always stated first for pH 4.5 followed by pH 6.0. Example: *Decrease of substrate affinity from pH 3.0 to 4.5 and 6.0 retaining x and y %, respectively.* To reduce the amount of redundancy, the term "*from pH 3.0 to 4.5 and 6.0 (...), respectively.*" was omitted in the following.

WT. Decrease of substrate affinity retaining 74 and 10 %. Decrease of catalytic turnover retaining 63 and 30 %. Decrease of catalytic efficiency retaining 46 and 2.9 %.

I424G. Weak to moderate decrease of substrate affinity retaining 66 and 10 %. Weak decrease of catalytic turnover retaining 99.2 and 57.2 %, that is, it shows the weakest decrease of all tested variants. Weak decrease of catalytic efficiency retaining 65.3 and 5.7 %, that is, it shows the weakest and second weakest decrease at pH 4.5 and 6.0, respectively, of all tested variants.

T383I/A180D. Moderate to weak decrease of substrate affinity retaining 42 and 16 %, that is, it shows weakest decrease at pH 6.0 of all tested variants. Moderate decrease of catalytic turnover retaining 74 and 42 %. Moderate to weak decrease of catalytic efficiency retaining 31 and 6.8 %.

L499F-containing variants. Moderate decrease of substrate affinity retaining ~45 and 5.5 %. Weak decrease of catalytic turnover retaining ~87 and 52 %. Moderate decrease of catalytic efficiency retaining ~40 and ~3 %, respectively.

W367F. Moderate decrease of substrate affinity retaining 49 and 8 %. Weak to moderate decrease of catalytic turnover retaining 86 and 35 %. Moderate decrease of catalytic efficiency retaining 42 and 3.0 %.

T383I/I424G/L499M and L499M. Strong decrease of substrate affinity retaining ~35 and 3 %. Moderate to weak decrease of catalytic turnover retaining ~70 and 53 %. Strong decrease of catalytic efficiency retaining ~24 and 1.5 %.

T383I/I491N. Strong to moderate decrease of substrate affinity retaining 18.9 and 10.5 %. Weak to strong decrease of catalytic turnover from retaining 84 and 24.8 %. Strong to moderate decrease of catalytic efficiency retaining 15.8 and 2.6 %.

T383I/D236E/I424G. Strong decrease of substrate affinity retaining 34 and 6 %. Weak decrease of catalytic turnover retaining 91.3 and 29.7 %. Strong decrease of catalytic efficiency retaining 31 and 1.9 %.

T383I/I424M. Weak to strong decrease of substrate affinity retaining 56 and 8.2 %. Moderate to strong decrease of catalytic turnover retaining 69 and 27.9 %. Moderate to strong decrease of catalytic efficiency retaining 39 and 2.3 %.

D236E. Strong decrease of substrate affinity retaining 40 and 3.9 %. Moderate decrease of catalytic turnover retaining 67 and 32 %. Strong decrease of catalytic efficiency retaining 27.2 and 1.3 %.

In the following, the absolute behaviour of kinetic constants relative to the WT for the substrate ABTS is described for each variant. Percentages of retention or yields are always related to values of the WT set to 100 % and are always stated for pH 3.0 first, followed by pH 4.5 and 6.0. Example: *High substrate affinities retaining / yielding x, y and z % of the wild-type at pH 3.0, 4.5 and 6.0, respectively.* To reduce the amount of redundancy, the term "*of the wild-type at pH 3.0, 4.5 and 6.0, respectively.*" was omitted in the following.

L499F. High to moderate substrate affinities retaining 101, 65 and 61 %. High turnover numbers yielding 97, 134 and 176, that is, it shows the highest turnover numbers at pH 4.5 and 6.0 of all tested variants. High catalytic efficiencies retaining 97, 87 and 107 %.

I424G. High substrate affinities yielding 121, 108 and 120 %, that is, it shows the highest substrate affinity at pH 4.5 of all tested variants. High catalytic turnover yielding 77, 121 and 149 %, that is, it shows the third and second highest turnover numbers at pH 4.5 and 6.0, respectively, of all tested variants. High catalytic efficiencies yielding 91, 131 and 178 %, that is, it shows the highest efficiencies at pH 4.5 and 6.0 of all tested variants.

D236E. High to moderate substrate affinities retaining 98, 53 and 38 %. High catalytic turnover yielding 118, 125 and 126 %, that is, it shows the highest, second highest and third highest turnover numbers at pH 3.0, 4.5 and 6.0, respectively, of all tested variants. High to moderate catalytic efficiencies yielding 114, 68 and 48 %, that is, it shows the highest efficiency at pH 3.0 of all tested variants.

T383I/I424M. High substrate affinities yielding 108, 82 and 89 %. Moderate catalytic turnover retaining 67, 73 and 63 %. High to moderate catalytic efficiencies retaining 72, 60 and 56 %.

T383I/A180D/L499F. Moderate to low substrate affinities retaining 48, 27 and 24 %. Moderate to high turnover numbers retaining 49, 68 and 90 %. Moderate to low efficiencies retaining 23, 18 and 22 %.

L499M. Low substrate affinities retaining 37, 16 and 12 %. Moderate to high turnover numbers retaining 53, 59 and 91 %. Low catalytic efficiencies retaining 19, 10 and 11 %.

T383I/I424M/L499F. High to moderate substrate affinities retaining 82, 45 and 40 %. Moderate turnover numbers retaining 50, 56 and 67 %. Moderate catalytic efficiencies retaining 32, 25 and 27 %.

W367F. High substrate affinities yielding 159, 105 and 133 %, that is, it shows the highest substrate affinities at pH 3.0 and 6.0 of all tested variants. Low turnover numbers retaining 37, 50 and 44 %. Moderate to high catalytic efficiency retaining 58, 52 and 58 %.

T383I/D236E/I424G. Moderate substrate affinities retaining 69, 32 and 42 %. Low turnover numbers retaining 30, 43 and 30 %. Low catalytic efficiencies retaining 21, 14 and 13 %.

T383I/I424G/L499M. Low substrate affinities retaining 19.3, 9.3 and 4.4 %, that is, it shows the lowest substrate affinity at pH 6.0 of all tested variants. Low to moderate turnover numbers retaining 26, 28 and 48 %. Low catalytic efficiencies retaining 5.0, 2.6 and 2.1 %.

T383I/A180D. Low substrate affinities retaining 11.7, 6.6 and 19.0 %. Low turnover numbers retaining 18, 20 and 25 %. Low catalytic efficiencies retaining 2.1, 1.4 and 4.8 %.

T383I/I491N. Low substrate affinities retaining 6.2, 1.6 and 6.4 %, that is, it shows the lowest substrate affinities at pH 3.0 and 4.5 of all tested variants. Low catalytic turnover retaining 3.1, 4.1 and 2.6 %, that is, it yields the lowest turnover numbers at pH 3.0, 4.5 and 6.0 of all tested variants. Low catalytic efficiencies retaining 0.19, 0.06 and 0.16 %, that is, it shows the lowest catalytic efficiencies at pH 3.0, 4.5 and 6.0 of all tested variants.

Effects of mutations

L499M. Bacterial laccases have a conserved Met at this position, and it has been shown that the *Ba*L variant L499M has lower T1 redox potential (580 mV *vs*. SHE [2]), similar to bacterial laccases. The decrease of the T1 redox potential seems to be responsible for the observed changes in the kinetic constants of **L499M**. Whereas k_{cat} for the substrate with the higher oxidation potential ($ABTS/ABTS^{+\bullet}$, 672 mV *vs*. SHE [3]) is ~1.9- (pH 3.0) – 1.4-fold (pH 6.0) lower compared to the wild-type *Ba*L, the k_{cat} for DMP (oxidation potential 580 mV *vs*. SHE [4]) increases at pH 4.5 and 6.0, presumably because of the faster electron transfer between the T1 and T2/T3 copper sites due to the higher potential difference. L499M showed a reduced activity towards ABTS and DMP below pH 6.0 and a bell- or plateau-shaped pH profile (pH-type I) [2].

I424M. Met at this position has no effect on ABTS binding, but clashes with the preferred binding position of DMP, which explains the much higher K_M value. The importance of Ile for the positioning of DMP in the substrate binding pocket can be drawn from the docking results shown in Fig. 2. Additionally, to the steric effects, the exchange of Ile into Met very likely reduces its T1 copper redox potential. This position is little conserved in laccases. A multiple sequence alignment shows that ascomycete laccases tend to have a Leu, basidiomycete ones an Ala or Gly, plant laccases and L-ascorbate oxidases a negatively charged residue (Asp, Glu), bacterial laccases a

Met or Phe, and bacterial CotA also a Gly at this position (Supplemental Fig. S5). Structural examination reveals that the loop hosting this position is extended by at least one residue in ascomycete laccases compared to those from basidiomycetes, forcing the ribbon to move away from the T1 copper.

Ile491 is one of the two axial, non-coordinating ligands of the T1 copper and is fully conserved in all fungal laccases. It faces the substrate-binding site and shields the copper atom from water molecules thereby maintaining the high redox potential of the T1 copper [5]. It is reported that a mutation of this amino acid to alanine lead to an almost inactive variant [5]. It was therefore surprising to identify variant I491N as a positive clone by differential screening in the random directed evolution approach, having a higher relative activity at pH 6.5 than the wild-type *Ba*L (77% *vs.* 30%; Supplemental Table S7, S9, and S11, Supplemental Fig. S3). However, the increased relative activity at pH 6.5 is achieved at the cost of a much lower specific activity at pH 6.5 (27 vs 0.6 U/mg). This mutation dramatically increases the K_M for DMP and ABTS and strongly decreases the k_{cat} for both substrates. The I491N side chain in the modelled structure (based on *Ba*L 3SQR) is oriented towards the copper ion and might coordinate the T1 copper by an unshared pair of electrons via the γ-oxygen of the γ-amide group similar to low redox potential blue copper proteins like umecyanin [6], which presumably reduces the T1 redox potential and interferes with the substrate binding.

Ala180 is buried at the edge of the substrate-binding site, 13 Å away from the T1 copper ion. Variant A180D was identified by a random directed evolution approach. The bulkier aspartate seems to affect the folding of the α-helix $_{173\text{-}183}$ and results in conformational changes. By introducing the charge into the binding site, this mutation acts similarly as the mutation D236E as both variants show pH profiles in which the activity is better retained at higher pH. However,

this effect is much lower for A180D, yet a pH-dependent protonation mechanism similar to D236E can be assumed. In contrast to D236E, negative effects on substrate binding and catalysis prevail in A180D (specific activity at pH 6.5 of 6.3 U/mg compared to 27 U/mg for *Ba*L and D236E). The distance of 10.5 Å of its γ-carboxyl group to the copper ion makes it unlikely that the T1 redox potential is affected.

Trp367 is close to the T1 copper. This Trp is strictly conserved in ascomycetous laccases, whereas Phe is conserved in basidiomycetous laccases at this position. In *Ba*L, the indole ring of W367 lies 5.5 Å away from the copper ion (edge-to-centre) and shields the copper ion from water molecules. In a homology model (SwissModel) of variant W367F Phe has moved closer to the copper ion (4.3 Å) but also shields a smaller area of the copper ion. The effect of this mutation on the kinetic constants for DMP is unfavourable. The K_M of W367F is reduced 2–3-fold for ABTS which however is accompanied by a three-fold reduced k_{cat}. Since W367 is not part of the substrate-binding site, no sterical reasons can be assumed for a reduced K_M, so this might be a kinetic effect connected to the decreased k_{cat} (Table 4).

Materials &Methods

Sequencing

PCR products were purified using the Illustra GFX PCR DNA and Gel Band Purification Kit (GE Healthcare, Little Chalfont, UK). Purified PCR products were sequenced using the primers pGAPfw2 (5'-CCC AAT TTT GGT-3′) and 3ZLHGAP1 (5'-GGC GCT ATT CAG-3′) by Microsynth (Balgach, CH).

Modelling

Homology models were done by using the online tool Swiss-Model. Point mutations were introduced into the BaL sequence (PDB entry 3SQR) and modelled using the original sequence as

template. The proposed rotamer for the point mutations was employed in further studies with PyMOL (DeLano Scientific, Palo Alto, CA, US).

Alignment

Multiple sequence and structure alignment was done by using the online tool PROMALS3D (http://prodata.swmed.edu/promals3d/promals3d.php,[7]). Representative sequences of ascomyceteous, basidiomyceteous, bacterial and plant laccases as well as sequences of laccase-like bacterial spore coat A proteins and plant L-ascorbate oxidases were compared.

Protein similarity

Protein sequence and structure similarities were calculated by using the online tool PDBeFold (http://www.ebi.ac.uk/msd-srv/ssm/,[8–11]). Sequence identities are given in % and structural similarities are given as the Root Mean Square Deviation (RMSD) presented in angstroms.

Supplemental Tables

Results

Generationof laccase variants

Supplemental Table S1: *Ba*L variants from two rounds of directed evolution. The first generation evolved the thermostable variant T383I, and the second generation the most promising – evaluated by relative activity – variants concerning improved activity at elevated pH. Fold-improvements relative to the wild-type concerning the depicted parameters are shown. t_{50} is the half-life in minutes defined as the time after which 50% relative activity after incubation at 55 °C are retained.

		fold impr. t_{50}	fold impr. pH 6.5	
	variant		DMP	ABTS
parent	*Ba*L wt	1	1	1
1st generation	T383I	2.5	1.1	1.0
2nd generation	T383I/A180D	n.d.	1.5	1.3
	T383I/I424M	n.d.	2.2	1.6
	T383I/I491N	n.d.	2.6	0.6

Increased Thermostability

Supplemental Table S2: Interface summary. Structural parameters of the interface between a 31-residues long loop-helix-loop motif of *Botrytis aclada* laccase hosting Thr383 (structure 1, selection range B) or the respective mutation Ile383 (structure 3, selection range B), respectively, and the whole *Ba*L without the specified motif (structure 2, selection range B) calculated by the online tool PDBePISA.

	Structure 1 T383 helix		Structure 2		Structure 3 I383 helix		Structure 2	
Selection range	B		A		B		A	
class	Protein		Protein		Protein		Protein	
symmetry operation	x,y,z		x,y,z		x,y,z		x,y,z	
symmetry ID	1_555		0_555		1_555		0_555	
Number of atoms								
interface	104	41.60%	147	3.70%	105	41.80%	147	3.70%
surface	188	75.20%	1831	46.60%	189	75.30%	1831	46.60%
total	250	100.00%	3931	100.00%	251	100.00%	3931	100.00%
Number of residues								
interface	24	77.40%	51	10.00%	24	77.40%	51	10.00%
surface	31	100.00%	446	87.80%	31	100.00%	446	87.80%
total	31	100.00%	508	100.00%	31	100.00%	508	100.00%
Solvent-accessible area, Å								
interface	1342.4	47.50%	1140.5	6.20%	1338	47.50%	1140.5	6.20%
total	2823.7	100.00%	18447.6	100.00%	2819.2	100.00%	18447.6	100.00%
Solvation energy, kcal/mol								
isolated structure	-19	100.00%	-480.7	100.00%	-19.8	100.00%	-480.7	100.00%
gain on complex formation	-12.9	67.90%	-7.1	1.50%	-13.6	68.90%	-7.1	1.50%
average gain	-9.3	48.80%	-4.9	1.00%	-9.7	49.10%	-4.9	1.00%
P-value	0.122		0.239		0.104		0.239	

Supplemental Table S3: Hydrogen bonds and distances between atoms of interfacing residues of a 31 residues long loop-helix-loop motif hosting Thr383 (structure 1) and the whole *Ba*L without the specified motif (structure 2).

##	Structure 1 T383 helix	Dist. [Å]	Structure 2
1	B:ASN 370[N]	2.95	A:GLN 356[O]
2	B:SER 373[OG]	3.07	A:GLU 497[O]
3	B:LEU 374[N]	3.16	A:GLY 498[O]
4	B:LEU 376[N]	2.92	A:SER 541[O]
5	B:TRP 378[NE1]	2.74	A:GLY 542[O]
6	B:THR 382[N]	3.06	A:GLY 104[O]
7	B:THR 382[N]	3.46	A:VAL 105[O]
8	B:THR 382[OG1]	3.43	A:GLY 104[O]
9	B:THR 382[OG1]	2.65	A:GLN 502[OE1]
**10**	**B:THR 383[N]**	**2.86**	**A:VAL 105[O]**
**11**	**B:THR 383[OG1]**	**2.73**	**A:VAL 105[O]**
**12**	**B:THR 383[OG1]**	**3.17**	**A:SER 484[OG]**
13	B:ASN 398[ND2]	2.92	A:ALA 500[O]
14	B:ASN 370[OD1]	2.87	A:GLN 356[N]
15	B:GLU 390[O]	2.69	A:GLN 507[NE2]
16	B:TYR 397[O]	2.83	A:GLN 502[NE2]
17	B:ASN 398[O]	3.46	A:VAL 400[N]
18	B:ASN 398[O]	3.06	A:GLN 502[N]

Supplemental Table S4: Structural and thermodynamic parameters of hydrogen bonding residues of a 31 residues long loop-helix-loop motif hosting Thr383 (structure 1) and the whole *Ba*L without the specified motif (structure 2). ASA, accessible surface area; BSA, buried surface area, ΔiG, solvation energy effect.

Structure 1 T383 helix	ASA [Å²]	BSA [Å²]	BSA [%]	ΔiG [kcal mol^{-1}]	Structure 2	ASA [Å²]	BSA [Å²]	BSA [%]	ΔiG [kcal mol^{-1}]
B:ASN 370	148.46	64.74	43.6	-0.47	A:GLY 104	72.65	72.65	100	0.36
B:SER 373	93.76	65.94	70.3	0.16	**A:VAL 105**	**29.86**	**29.86**	**100**	**-0.11**
B:LEU 374	103.15	100.46	97.4	0.97	A:GLN 356	95.93	31.84	33.2	-0.11
B:LEU 376	85.07	80.94	95.1	0.77	A:VAL 400	58.34	37.73	64.7	-0.08
B:TRP 378	240.78	212.67	88.3	2.16	**A:SER 484**	**16.76**	**16.52**	**98.6**	**0.20**
B:THR 382	45.76	45.76	100.0	0.29	A:GLU 497	6.76	0.83	12.3	0.00
**B:THR 383**	**67.32**	**67.32**	**100.0**	0.26	A:GLY 498	25.28	25.11	99.3	0.11
B:GLU 390	68.95	3.19	4.6	-0.04	A:ALA 500	36.65	36.65	100	-0.10
B:TYR 397	11.53	8.62	74.8	-0.07	A:GLN 502	66.94	66.94	100	-0.44
B:ASN 398	49.68	35.02	70.5	-0.16	A:GLN 507	111.43	87.72	78.7	-0.73
					A:SER 541	49.14	46.73	95.1	0.11
					A:GLY 542	16.60	16.60	100	0.19

Supplemental Table S5: Hydrogen bonds and distances between atoms of interfacing residues of a 31 residues long loop-helix-loop motif hosting Ile383 (structure 3) and the whole *Ba*L without the specified motif (structure 2).

##	Structure 3 I383 helix	Dist. [Å]	Structure 2
1	B:ASN 370[N]	2.95	A:GLN 356[O]
2	B:SER 373[OG]	3.07	A:GLU 497[O]
3	B:LEU 374[N]	3.16	A:GLY 498[O]
4	B:LEU 376[N]	2.92	A:SER 541[O]
5	B:TRP 378[NE1]	2.74	A:GLY 542[O]
6	B:THR 382[N]	3.06	A:GLY 104[O]
7	B:THR 382[N]	3.46	A:VAL 105[O]
8	B:THR 382[OG1]	3.43	A:GLY 104[O]
9	B:THR 382[OG1]	2.65	A:GLN 502[OE1]
10	**B:ILE 383[N]**	2.90	**A:VAL 105[O]**
11	B:ASN 398[ND2]	2.92	A:ALA 500[O]
12	B:ASN 370[OD1]	2.87	A:GLN 356[N]
13	B:GLU 390[O]	2.69	A:GLN 507[NE2]
14	B:TYR 397[O]	2.83	A:GLN 502[NE2]
15	B:ASN 398[O]	3.46	A:VAL 400[N]
16	B:ASN 398[O]	3.06	A:GLN 502[N]

Supplemental Table S6: Structural and thermodynamic parameters of hydrogen bonding residues of a 31 residues long loop-helix-loop motif hosting Ile383 (structure 3) and the whole *Ba*L without the specified motif (structure 2). ASA, accessible surface area; BSA, buried surface area, ΔiG, solvation energy effect.

Structure 3 I383 helix	ASA [Å²]	BSA [Å²]	BSA [%]	ΔiG [kcal mol^{-1}]	Structure 2	ASA [Å²]	BSA [Å²]	BSA [%]	ΔiG [kcal mol^{-1}]
B:ASN 370	148.46	64.74	43.6	-0.47	A:GLY 104	72.65	72.65	100.0	0.36
B:SER 373	93.76	65.94	70.3	0.16	**A:VAL 105**	**29.86**	**29.86**	**100.0**	**-0.11**
B:LEU 374	103.15	100.46	97.4	0.97	A:GLN 356	95.93	31.84	33.2	-0.11
B:LEU 376	85.07	80.94	95.1	0.77	A:VAL 400	58.34	37.73	64.7	-0.08
B:TRP 378	240.78	212.67	88.3	2.16	A:GLU 497	6.76	0.83	12.3	0.00
B:THR 382	46.60	46.60	100.0	0.30	A:GLY 498	25.28	25.11	99.3	0.11
**B:ILE 383**	**82.83**	**82.83**	**100.0**	**1.29**	A:ALA 500	36.65	36.65	100.0	-0.10
B:GLU 390	68.95	3.19	4.6	-0.04	A:GLN 502	66.94	66.94	100.0	-0.44
B:TYR 397	11.53	8.62	74.8	-0.07	A:GLN 507	111.43	87.72	78.7	-073
B:ASN 398	49.68	35.02	70.5	-0.16	A:SER 541	49.14	46.73	95.1	0.11
					A:GLY 542	16.60	16.60	100.0	0.19

Shifting laccase activity towards neutral pH

Supplemental Table S7: Relative activities plus standard deviation and fold improvement / deterioration related to the wild-type of depicted *Ba*L variants towards ABTS at indicated pH values.

	Relative activity, %						fold improvement					
	pH						pH					
variant	3.0	4.0	5.0	6.0	7.0	7.5	3.0	4.0	5.0	6.0	7.0	7.5
wt	96 ± 3	78 ± 2	50 ± 1	32 ± 2	6.7 ± 0.4	2.12 ± 0.19	-	-	-	-	-	-
D236E	100 ± 0	93 ± 0	82 ± 1	49 ± 1	7.4 ± 0.2	2.23 ± 0.30	1.0	1.2	1.6	1.6	1.1	1.1
T383I	97 ± 2	71 ± 3	51 ± 3	32 ± 1	7.0 ± 0.8	2.10 ± 0.50	1.0	0.9	1.0	1.0	1.0	1.0
W367F	97 ± 4	82 ± 4	60 ± 5	38 ± 8	8.8 ± 0.6	3.69 ± 0.22	1.0	1.1	1.2	1.2	1.3	1.7
I424G	90 ± 4	99 ± 4	79 ± 4	56 ± 3	18.9 ± 3.0	7.51 ± 0.37	0.9	1.3	1.6	1.8	2.8	3.5
L499F	100 ± 0	95 ± 4	88 ± 4	67 ± 3	24.8 ± 1.0	12.16 ± 0.99	1.0	1.2	1.8	2.1	3.7	5.7
L499M	100 ± 0	96 ± 2	79 ± 1	46 ± 5	10.9 ± 0.3	4.94 ± 0.31	1.0	1.2	1.6	1.5	1.6	2.3
T383I/A180D	95 ± 3	78 ± 4	54 ± 5	35 ± 1	7.4 ± 1.8	1.18 ± 0.46	1.0	1.0	1.1	1.1	1.1	0.6
T383I/I424M	98 ± 5	92 ± 5	69 ± 6	47 ± 3	14.1 ± 1.5	6.23 ± 0.60	1.0	1.2	1.4	1.5	2.1	2.9
T383I/I491N	100 ± 0	82 ± 4	46 ± 2	19.4 ± 0.7	8.8 ± 1.0	5.15 ± 0.69	1.0	1.1	0.9	0.6	1.3	2.4
T383I/D236E/I424G	100 ± 0	92 ± 2	76 ± 3	46 ± 4	10.7 ± 1.4	3.56 ± 1.12	1.0	1.2	1.5	1.5	1.6	1.7
T383I/A180D/L499F	100 ± 0	93 ± 3	78 ± 2	48 ± 2	4.1 ± 0.5	0.98 ± 0.41	1.0	1.2	1.6	1.5	0.6	0.5
T383I/I424G/L499M	100 ± 0	84 ± 2	58 ± 1	22.4 ± 1.3	4.0 ± 0.4	1.52 ± 0.34	1.0	1.1	1.2	0.7	0.6	0.7
T383I/I424M/L499F	100 ± 0	92 ± 9	78 ± 4	51 ± 3	15.1 ± 0.7	5.34 ± 0.20	1.0	1.2	1.6	1.6	2.3	2.5

Supplemental Table S8: Specific activities plus standard deviation and fold improvement / deterioration related to the wild-type of depicted *Ba*L variants towards ABTS at indicated pH values.

	specific activity, U mg^{-1}						fold improvement					
	pH						pH					
variant	3.0	4.0	5.0	6.0	7.0	7.5	3.0	4.0	5.0	6.0	7.0	7.5
wt	124 ± 3	103 ± 0	66 ± 1	41 ± 1	8.8 ± 0.5	2.80 ± 0.28	-	-	-	-	-	-
D236E	115 ± 1	107 ± 0	94 ± 2	56 ± 1	8.5 ± 0.2	2.58 ± 0.32	0.9	1.0	1.4	1.4	1.0	0.9
T383I	119 ± 5	88 ± 0	64 ± 2	40 ± 2	8.5 ± 0.7	2.57 ± 0.57	1.0	0.9	1.0	1.0	1.0	0.9
W367F	53 ± 2	44 ± 0	33 ± 1	20.4 ± 3.4	4.7 ± 0.2	2.05 ± 0.16	0.4	0.4	0.5	0.5	0.5	0.7
I424G	86 ± 3	96 ± 0	77 ± 6	55 ± 4	18.2 ± 2.9	7.33 ± 0.67	0.7	0.9	1.2	1.3	2.1	2.6
L499F	109 ± 5	104 ± 0	96 ± 6	72 ± 4	27.0 ± 1.4	13.37 ± 1.19	0.9	1.0	1.5	1.8	3.1	4.8
L499M	61 ± 1	59 ± 0	48 ± 2	29.0 ± 3.2	6.6 ± 0.1	3.08 ± 0.27	0.5	0.6	0.7	0.7	0.8	1.1
T383I/A180D	22.6 ± 0.4	18.8 ± 0.0	12.9 ± 0.4	8.4 ± 0.3	1.80 ± 0.56	0.29 ± 0.12	0.2	0.2	0.2	0.2	0.2	0.1
T383I/I424M	76 ± 7	71 ± 0	54 ± 7	37 ± 3	10.9 ± 1.5	4.87 ± 0.49	0.6	0.7	0.8	0.9	1.2	1.7
T383I/I491N	4.7 ± 0.2	3.9 ± 0.0	2.19 ± 0.03	0.94 ± 0.09	0.42 ± 0.06	0.25 ± 0.05	0.0	0.0	0.0	0.0	0.1	0.1
T383I/D236E/I424G	38 ± 1	35 ± 0	29.2 ± 0.9	17.6 ± 1.4	4.1 ± 0.5	1.367 ± 0.415	0.3	0.3	0.5	0.4	0.5	0.5
T383I/A180D/L499F	55 ± 2	51 ± 0	43 ± 1	27.1 ± 1.3	2.26 ± 0.20	0.553 ± 0.227	0.4	0.5	0.7	0.7	0.3	0.2
T383I/I424G/L499M	11.5 ± 0.7	10.4 ± 1.1	10.0 ± 0.8	7.7 ± 0.9	2.92 ± 0.36	0.967 ± 0.238	0.1	0.1	0.2	0.2	0.3	0.3
T383I/I424M/L499F	56 ± 5	52 ± 0	44 ± 4	28.6 ± 1.8	8.4 ± 1.0	2.98 ± 0.239	0.5	0.5	0.7	0.7	1.0	1.1

Supplemental Table S9: Relative activities plus standard deviation and fold improvement / deterioration related to the wild-type of depicted *Ba*L variants towards DMP at indicated pH values.

	Relative activity, %						fold improvement					
	pH						pH					
variant	3.0	4.0	5.0	6.0	7.0	7.5	3.0	4.0	5.0	6.0	7.0	7.5
wt	84 ± 1	60 ± 2	47 ± 2	39 ± 1	13.3 ± 1.0	5.02 ± 0.45	-	-	-	-	-	-
D236E	100 ± 0	96 ± 5	94 ± 6	84 ± 5	34 ± 1	14.84 ± 1.25	1.2	1.6	2.0	2.2	2.6	3.0
T383I	88 ± 3	68 ± 3	53 ± 2	42 ± 2	13.7 ± 0.7	4.30 ± 0.27	1.1	1.1	1.1	1.1	1.0	0.9
W367F	98 ± 4	78 ± 1	65 ± 2	55 ± 2	13.0 ± 2.9	3.85 ± 2.01	1.2	1.3	1.4	1.4	1.0	0.8
I424G	84 ± 6	96 ± 4	86 ± 4	80 ± 7	23.6 ± 3.5	15.71 ± 0.51	1.0	1.6	1.8	2.1	1.8	3.1
L499F	100 ± 0	98 ± 3	89 ± 4	82 ± 3	25.5 ± 1.6	13.62 ± 1.82	1.2	1.7	1.9	2.1	1.9	2.7
L499M	94 ± 1	100 ± 0	98 ± 2	91 ± 3	32 ± 6	15.29 ± 5.86	1.1	1.7	2.1	2.4	2.4	3.0
T383I/A180D	100 ± 0	91 ± 2	77 ± 5	64 ± 2	22.3 ± 3.9	6.80 ± 1.90	1.2	1.5	1.6	1.7	1.7	1.4
T383I/I424M	77 ± 2	97 ± 1	100 ± 2	88 ± 3	39 ± 2	18.09 ± 1.39	0.9	1.6	2.1	2.3	2.9	3.6
T383I/I491N	72 ± 4	100 ± 0	87 ± 3	81 ± 1	64 ± 2	49.27 ± 4.04	0.9	1.7	1.8	2.1	4.8	9.8
T383I/D236E/I424G	100 ± 0	90 ± 14	87 ± 8	68 ± 10	26.0 ± 2.8	8.41 ± 1.70	1.2	1.5	1.8	1.8	2.0	1.7
T383I/A180D/L499F	100 ± 0	98 ± 7	89 ± 5	78 ± 6	18.1 ± 1.5	1.029 ± 1.08	1.2	1.6	1.9	2.0	1.4	0.2
T383I/I424G/L499M	21.8 ± 3.5	65 ± 5	100 ± 4	95 ± 3	65 ± 3	26.49 ± 1.40	0.3	1.1	2.1	2.5	4.9	5.3
T383I/I424M/L499F	57 ± 6	79 ± 5	98 ± 6	92 ± 6	54 ± 5	22.55 ± 2.00	0.7	1.3	2.1	2.4	4.1	4.5

Supplemental Table S10: Specific activities plus standard deviation and fold improvement / deterioration related to the wild-type of depicted *Ba*L variants towards DMP at indicated pH values.

	specific activity, U mg^{-1}						fold improvement					
	pH						pH					
variant	3.0	4.0	5.0	6.0	7.0	7.5	3.0	4.0	5.0	6.0	7.0	7.5
wt	41 ± 2	29.0 ± 0.0	23.0 ± 1.0	18.6 ± 0.8	6.4 ± 0.4	2.45 ± 0.26	-	-	-	-	-	-
D236E	32 ± 2	34 ± 0	33 ± 1	30 ± 0	12.0 ± 1.1	5.25 ± 0.50	0.8	1.2	1.4	1.6	1.9	2.1
T383I	34 ± 1	27.0 ± 0.0	21.1 ± 1.1	16.4 ± 0.7	5.3 ± 0.3	1.73 ± 0.15	0.8	0.9	0.9	0.9	0.8	0.7
W367F	16.6 ± 0.5	13.3 ± 0.0	11.0 ± 0.2	9.5 ± 0.3	2.18 ± 0.50	0.810 ± 0.147	0.4	0.5	0.5	0.5	0.3	0.3
I424G	27.4 ± 3.4	31 ± 0	26.7 ± 0.3	25.0 ± 1.4	7.6 ± 0.7	5.00 ± 0.27	0.7	1.1	1.2	1.3	1.2	2.0
L499F	36 ± 1	35 ± 0	32 ± 1	29.3 ± 0.9	9.1 ± 0.5	4.82 ± 0.76	0.9	1.2	1.4	1.6	1.4	2.0
L499M	19.1 ± 0.3	20.2 ± 0.0	19.8 ± 0.4	18.2 ± 0.5	6.4 ± 1.1	3.09 ± 1.19	0.5	0.7	0.9	1.0	1.0	1.3
T383I/A180D	6.3 ± 0.2	5.7 ± 0.0	5.0 ± 0.3	4.1 ± 0.2	1.41 ± 0.23	0.431 ± 0.115	0.2	0.2	0.2	0.2	0.2	0.2
T383I/I424M	12.7 ± 0.4	16.4 ± 0.0	16.8 ± 0.3	14.8 ± 0.6	6.4 ± 0.4	3.01 ± 0.28	0.3	0.6	0.7	0.8	1.0	1.2
T383I/I491N	0.71 ± 0.04	0.98 ± 0.00	0.86 ± 0.02	0.80 ± 0.01	0.63 ± 0.02	0.484 ± 0.036	0.0	0.0	0.0	0.0	0.1	0.2
T383I/D236E/I424G	11.5 ± 0.7	10.4 ± 1.1	10.0 ± 0.8	7.7 ± 0.9	2.92 ± 0.36	0.967 ± 0.239	0.3	0.4	0.4	0.4	0.5	0.4
T383I/A180D/L499F	15.9 ± 0.8	15.5 ± 1.5	14.2 ± 0.7	12.4 ± 1.0	2.83 ± 0.24	0.165 ± 0.171	0.4	0.5	0.6	0.7	0.4	0.1
T383I/I424G/L499M	1.57 ± 0.22	4.7 ± 0.3	7.3 ± 0.4	6.9 ± 0.4	4.8 ± 0.2	1.916 ± 0.157	0.0	0.2	0.3	0.4	0.7	0.8
T383I/I424M/L499F	7.3 ± 0.8	10.2 ± 0.4	12.6 ± 0.7	11.8 ± 1.1	6.9 ± 0.7	2.85 ± 0.216	0.2	0.4	0.6	0.6	1.1	1.2

Supplemental Table S11: Type of pH effects, pH-optima and specifications of depicted *Ba*L variants in terms of relative activity. Residual activities in % show the remaining activity at a given pH related to the highest observed activity (100 %) of the same variant at its pH optimum.

	ABTS								DMP							
			res. act. at pH %			fold impr. at pH					res. act. at pH %			fold impr. at pH		
variant	pH-type	pH opt.	2.5	5.5	6.5	2.5	5.5	6.5	pH-type	pH opt.	2.5	5.5	6.5	2.5	5.5	6.5
wt	-	2.5	100	43	20.6	-	-	-	-	2.5	100	44	29.9	-	-	-
T383I	-	2.5	100	41	20.1	1.00	0.97	0.97	-	2.5	100	49	32	1.00	1.10	1.07
D236E	II	3.0	98	72	23.6	1.00	1.67	1.14	II+III	2.5	100	92	64	0.92	2.10	2.20
W367F	-	2.5	100	53	19.0	1.00	1.23	0.93	II	2.5	100	62	26.2	1.00	1.39	0.90
I424G	(I)+II	3.5	86	69	42	0.87	1.60	2.03	I+II+III	3.5	76	83	51	0.78	1.93	1.72
L499F	II+III	3.0	100	81	46	1.00	1.87	2.19	II+III	3.0	96	89	59	0.97	2.03	1.99
L499M	I+II	3.0	99	67	24.8	1.00	1.55	1.17	II	4.0	81	98	63	0.81	2.21	2.10
T383I/A180D	-	2.5	100	44	26.3	1.00	1.01	1.29	II	3.0	92	72	47	0.91	1.58	1.54
T383I/I424M	II	3.5	94	59	33	0.96	1.36	1.57	I+III	4.5	64	95	65	0.65	2.15	2.18
T383I/I491N	-	3 0	98	29.5	13.5	0.97	0.67	0.64	I+II+III	4.0	47	87	77	0.47	1.96	2.60
T383I/D236E/I424G	II	3.0	86	63	25.6	0.86	1.46	1.24	II	3.0	91	81	45	0.92	1.84	1.55
T383I/A180D/L499F	II	3.0	94	64	23.2	0.94	1.49	1.12	II	3.0	92	85	57	0.93	1.92	1.92
T383I/I424G/L499M	II	3.0	91	39	45	0.98	0.90	0.45	I	5.5	13.5	100	83	0.13	2.25	2.80
T383I/I424M/L499F	II	3.0	95	66	30	0.95	1.54	1.45	I+III	5.5	42	100	80	0.42	2.25	2.70

Kinetic characterisation of *Ba*L variants

Supplemental Table S12: Relative changes of kinetic constants of *Ba*L variants with ABTS as substrate. The table shows the fold increases (>1) or fold decrease (<1) of kinetic constants of different *Ba*L variants relative to pH 3.0 (upper half of the table) and relative to the wild-type (lower half of the table) for the substrate ABTS. A change of >1 of the *relative* K_M values indicates an improvement and thus a decrease in the *absolute* K_M value.

		fold change								
		K_M			k_{cat}			k_{cat}/K_M		
	variant \ pH	3.0	4.5	6.0	3.0	4.5	6.0	3.0	4.5	6.0
change relative to pH 3.0	I424G	1	0.658	0.100	1	0.992	0.572	1	0.653	0.057
	T383I/A180D	1	0.415	0.163	1	0.736	0.416	1	0.306	0.068
	L499F	1	0.476	0.060	1	0.871	0.538	1	0.415	0.033
	T383I/A180D/L499F	1	0.412	0.051	1	0.878	0.546	1	0.362	0.028
	T383I/I424M/L499F	1	0.407	0.049	1	0.889	0.505	1	0.361	0.025
	L499M	1	0.328	0.032	1	0.707	0.511	1	0.232	0.016
	W367F	1	0.487	0.084	1	0.859	0.353	1	0.418	0.030
	T383I/I424G/L499M	1	0.358	0.023	1	0.673	0.546	1	0.241	0.013
	T383I/I491N	1	0.189	0.105	1	0.838	0.248	1	0.158	0.026
	T383I/D236E/I424G	1	0.341	0.061	1	0.913	0.297	1	0.312	0.018
	WT	1	0.739	0.100	1	0.634	0.298	1	0.464	0.030
	T383I/I424M	1	0.560	0.082	1	0.690	0.280	1	0.387	0.023
	T383I	1	0.524	0.066	1	0.656	0.307	1	0.343	0.020
	D236E	1	0.403	0.039	1	0.675	0.318	1	0.272	0.013
change relative to the wild-type	L499F	1.010	0.651	0.609	0.973	1.336	1.759	0.973	0.869	1.071
	I424G	1.212	1.079	1.203	0.776	1.214	1.492	0.932	1.310	1.796
	D236E	0.977	0.532	0.384	1.178	1.253	1.259	1.139	0.667	0.484
	WT	1	1	1	1	1	1	1	1	1
	T383I	1.377	0.976	0.904	0.837	0.865	0.864	1.142	0.845	0.781
	T383I/I424M	1.082	0.820	0.886	0.675	0.734	0.633	0.723	0.602	0.561
	T383I/A180D/L499F	0.481	0.268	0.245	0.491	0.680	0.900	0.234	0.182	0.220
	L499M	0.365	0.162	0.117	0.529	0.590	0.908	0.191	0.096	0.106
	T383I/I424M/L499F	0.819	0.451	0.399	0.396	0.555	0.672	0.322	0.250	0.268
	W367F	1.595	1.051	1.330	0.368	0.498	0.436	0.581	0.524	0.580
	T383I/D236E/I424G	0.689	0.318	0.419	0.301	0.433	0.300	0.205	0.138	0.126
	T383I/I424G/L499M	0.193	0.093	0.044	0.263	0.279	0.482	0.050	0.026	0.021
	T383I/A180D	0.117	0.066	0.190	0.180	0.209	0.251	0.021	0.014	0.048
	T383I/I491N	0.062	0.016	0.064	0.031	0.041	0.026	0.002	0.001	0.002

Supplemental Table S13: Relative changes of kinetic constants of *Ba*L variants with DMP as substrate. The table shows the fold increases (>1) or fold decrease (<1) of kinetic constants of different *Ba*L variants relative to pH 3.0 (upper half of the table) and relative to the wild-type (lower half of the table) for the substrate DMP. A change of >1 of the *relative* K_M values indicates an improvement and thus a decrease in the *absolute* K_M value.

		fold change								
		K_M			k_{cat}			k_{cat}/K_M		
	variant \ pH	3.0	4.5	6.0	3.0	4.5	6.0	3.0	4.5	6.0
change relative to pH 3.0	T383I/I424G/L499M	1	9.261	13.313	1	1.256	1.694	1	11.700	22.686
	T383I/A180D	1	3.585	3.791	1	1.307	0.893	1	4.686	3.352
	L499M	1	6.101	10.285	1	1.085	0.925	1	6.810	9.787
	T383I/I424M/L499F	1	6.861	8.550	1	0.963	0.865	1	6.521	7.305
	T383I/D236E/I424G	1	10.574	13.566	1	0.849	0.590	1	8.973	7.998
	L499F	1	9.295	15.324	1	0.812	0.734	1	7.558	11.259
	T383I/I424M	1	4.304	10.495	1	1.055	0.634	1	5.165	7.569
	T383I	1	12.765	12.765	1	0.683	0.371	1	8.710	4.731
	D236E	1	4.250	3.643	1	0.851	0.584	1	3.617	2.130
	I424G	1	10.531	23.319	1	0.725	0.554	1	7.593	12.861
	W367F	1	7.382	10.410	1	0.664	0.341	1	4.910	3.554
	T383I/A180D/L499F	1	5.579	5.256	1	0.623	0.496	1	3.477	2.608
	WT	1	7.867	13.111	1	0.577	0.404	1	4.528	5.290
	T383I/I491N	1	3.619	2.734	1	0.561	0.646	1	2.044	1.779
change relative to wild-type	D236E	4.627	2.500	1.286	0.906	1.338	1.311	4.186	3.344	1.686
	L499F	0.416	0.492	0.486	1.007	1.419	1.829	0.418	0.698	0.890
	WT	1	1	1	1	1	1	1	1	1
	T383I	1.088	1.224	1.059	0.837	0.991	0.768	0.910	1.750	0.813
	I424G	0.056	0.074	0.099	0.911	1.145	1.250	0.051	0.085	0.124
	L499M	0.074	0.057	0.058	0.554	1.043	1.268	0.040	0.059	0.073
	T383I/I424M	0.036	0.020	0.029	0.532	0.974	0.835	0.017	0.019	0.024
	W367F	0.581	0.545	0.462	0.420	0.483	0.354	0.243	0.263	0.163
	T383I/A180D/L499F	0.371	0.263	0.149	0.427	0.462	0.524	0.158	0.121	0.078
	T383I/D236E/I424G	0.328	0.441	0.340	0.276	0.406	0.402	0.090	0.179	0.137
	T383I/I424M/L499F	0.042	0.036	0.027	0.228	0.380	0.488	0.010	0.014	0.013
	T383I/I424G/L499M	0.006	0.007	0.006	0.129	0.282	0.543	0.001	0.002	0.003
	T383I/A180D	0.692	0.315	0.200	0.082	0.185	0.180	0.056	0.058	0.036
	T383I/I491N	0.023	0.011	0.005	0.028	0.027	0.045	0.001	0.000	0.000

Principal Component Analysis

DMP

Supplemental Table S14: Correlation Matrix DMP

	K_M pH 3.0	K_M pH 4.5	K_M pH 6.0	k_{cat} pH 3.0	k_{cat} pH 4.5	k_{cat} pH 6.0	k_{cat}/K_M pH 3.0	k_{cat}/K_M pH 4.5	k_{cat}/K_M pH 6.0
K_M pH 3.0	1	0.96607	0.7971	0.41857	0.42643	0.29676	0.98801	0.94085	0.84828
K_M pH 4.5	0.96607	1	0.92212	0.5393	0.49009	0.33748	0.9628	0.97159	0.9236
K_M pH 6.0	0.7971	0.92212	1	0.66075	0.53348	0.35738	0.80531	0.90655	0.92457
k_{cat} pH 3.0	0.41857	0.5393	0.66075	1	0.93764	0.85325	0.49008	0.58638	0.73934
k_{cat} pH 4.5	0.42643	0.49009	0.53348	0.93764	1	0.94408	0.49903	0.56315	0.68189
k_{cat} pH 6.0	0.29676	0.33748	0.35738	0.85325	0.94408	1	0.37937	0.41499	0.585
k_{cat}/K_M pH 3.0	0.98801	0.9628	0.80531	0.49008	0.49903	0.37937	1	0.95719	0.88459
k_{cat}/K_M pH 4.5	0.94085	0.97159	0.90655	0.58638	0.56315	0.41499	0.95719	1	0.93515
k_{cat}/K_M pH 6.0	0.84828	0.9236	0.92457	0.73934	0.68189	0.585	0.88459	0.93515	1

Supplemental Table S15: Eigenvalues of the Correlation Matrix DMP

	Eigenvalue	Percentage of Variance	Cumulative
1	6.74772	74.97%	74.97%
2	1.76327	19.59%	94.57%
3	0.33276	3.70%	98.26%
4	0.07704	0.86%	99.12%
5	0.03863	0.43%	99.55%
6	0.02527	0.28%	99.83%
7	0.00935	0.10%	99.93%
8	0.00437	0.05%	99.98%
9	0.00159	0.02%	100.00%

Supplemental Table S16: Descriptive Statistics DMP

	N analysis	N missing	Mean	Standard Deviation
K_M pH 3.0	14	0	0.02827	0.0507
K_M pH 4.5	14	0	0.16183	0.2252
K_M pH 6.0	14	0	0.20654	0.2427
k_{cat} pH 3.0	14	0	21.85464	14.73124
k_{cat} pH 4.5	14	0	17.40787	10.89014
k_{cat} pH 6.0	14	0	12.99828	8.52249
k_{cat}/K_M pH 3.0	14	0	9.07809	19.60301
k_{cat}/K_M pH 4.5	14	0	43.47687	76.17715
k_{cat}/K_M pH 6.0	14	0	33.70864	48.95398

Supplemental Table S17: Extracted Eigenvectors DMP

	Coefficients of PC1	Coefficients of PC2
K_M pH 3.0	0.34114	-0.29996
K_M pH 4.5	0.36176	-0.24951
K_M pH 6.0	0.34924	-0.13947
k_{cat} pH 3.0	0.29759	0.43679
k_{cat} pH 4.5	0.2879	0.48495
k_{cat} pH 6.0	0.24019	0.55709
k_{cat}/K_M pH 3.0	0.35352	-0.23819
k_{cat}/K_M pH 4.5	0.36801	-0.18613
k_{cat}/K_M pH 6.0	0.37582	-0.02503

ABTS

Supplemental Table S18: Correlation Matrix ABTS

	K_M pH 3.0	K_M pH 4.5	K_M pH 6.0	k_{cat} pH 3.0	k_{cat} pH 4.5	k_{cat} pH 6.0	k_{cat}/K_M pH 3.0	k_{cat}/K_M pH 4.5	k_{cat}/K_M pH 6.0
K_M pH 3.0	1	0.94477	0.91055	0.60913	0.63994	0.46453	0.80959	0.78213	0.70107
K_M pH 4.5	0.94477	1	0.96974	0.63102	0.65504	0.48965	0.82958	0.89086	0.83012
K_M pH 6.0	0.91055	0.96974	1	0.45661	0.5085	0.34423	0.69473	0.80859	0.78287
k_{cat} pH 3.0	0.60913	0.63102	0.45661	1	0.94798	0.84552	0.92276	0.80019	0.66971
k_{cat} pH 4.5	0.63994	0.65504	0.5085	0.94798	1	0.94586	0.88869	0.85542	0.79581
k_{cat} pH 6.0	0.46453	0.48965	0.34423	0.84552	0.94586	1	0.73181	0.74892	0.74864
k_{cat}/K_M pH 3.0	0.80959	0.82958	0.69473	0.92276	0.88869	0.73181	1	0.91037	0.78388
k_{cat}/K_M pH 4.5	0.78213	0.89086	0.80859	0.80019	0.85542	0.74892	0.91037	1	0.96562
k_{cat}/K_M pH 6.0	0.70107	0.83012	0.78287	0.66971	0.79581	0.74864	0.78388	0.96562	1

Supplemental Table S19: Eigenvalues of the Correlation Matrix ABTS

	Eigenvalue	Percentage of Variance	Cumulative
1	7.09895	78.88%	78.88%
2	1.29574	14.40%	93.27%
3	0.39035	4.34%	97.61%
4	0.15355	1.71%	99.32%
5	0.03898	0.43%	99.75%
6	0.01492	0.17%	99.92%
7	0.00624	0.07%	99.99%
8	0.00117	0.01%	100.00%
9	9.80E-05	0.00%	100.00%

Supplemental Table S20: Descriptive Statistics ABTS

	N analysis	N missing	Mean	Standard Deviation
K_M pH 3.0	14	0	0.25881	0.1588
K_M pH 4.5	14	0	0.13037	0.09608
K_M pH 6.0	14	0	0.01844	0.01437
k_{cat} pH 3.0	14	0	76.89461	46.17266
k_{cat} pH 4.5	14	0	59.08681	34.23432
k_{cat} pH 6.0	14	0	31.43799	19.57036
k_{cat}/K_M pH 3.0	14	0	240.84429	196.1027
k_{cat}/K_M pH 4.5	14	0	97.03651	88.60385
k_{cat}/K_M pH 6.0	14	0	6.69552	6.83888

Supplemental Table S21: Extracted Eigenvectors ABTS

	Coefficients of PC1	Coefficients of PC2
K_M pH 3.0	0.32256	-0.35037
K_M pH 4.5	0.34085	-0.35841
K_M pH 6.0	0.30479	-0.50076
k_{cat} pH 3.0	0.32385	0.36031
k_{cat} pH 4.5	0.34016	0.35591
k_{cat} pH 6.0	0.29655	0.4832
k_{cat}/K_M pH 3.0	0.35667	0.07843
k_{cat}/K_M pH 4.5	0.36567	-0.02411
k_{cat}/K_M pH 6.0	0.34268	-0.03619

Materials & Methods

PCR Protocols

Reaction setups

Supplemental Table S22: Reaction setup for error prone PCR.

Component	25 µL reaction	Final concentration
10x Mutazyme II buffer	2.5 µL	1x
0.5 – 1.0 µg template DNA	4.0 µL	0.02 – 0.04 µg µL^{-1}
10 µM pGAPfw2-primer	0.5 µL	0.2 µM
10 µM 3ZLHGAP1-primer	0.5 µL	0.2 µM
10 mM dNTP’s	0.5 µL	200 µM
Mutazyme II DNA polymerase	0.5 µL	-
Sterile di-H_2O	16.5 µL	-

Supplemental Table S23: Phusion master mix

Component	1875 µL	Final concentration
5x HF buffer	750 µL	2.5x
Phusion DNA polymerase	37.5 µL	-
10 mM dNTP’s	75 µL	400 µM
Sterile di-H_2O	1012.5 µL	-

Supplemental Table S24: Reaction setup for Phusion PCR.

Component	25 µL reaction	Final concentration
1.0 - 20 µg template DNA	1.0 µL	0.02 – 0.04 µg µL^{-1}
10 µM 5’-primer	1.0 µL	0.4 µM
10 µM 3’-primer	1.0 µL	0.4 µM
Phusion master mix	12.5 µL	
Sterile di-H_2O	9.5 µL	-

Supplemental Table S25: Reaction setup for Colony PCR

Component	25 µL reaction	Final concentration
Template DNA	10.0 µL	-
10 µM 5’-primer	1.0 µL	0.4 µM
10 µM 3’-primer	1.0 µL	0.4 µM
5x GoTaq buffer	5.0 µL	1x
10 mM dNTP’s	0.5	200 µM
GoTaq G2 Polymerase	0.5	-
Sterile di-H_2O	7.0 µL	-

Reaction conditions

Supplemental Table S26: Reaction conditions for error prone PCR

Step	Temp (°C)	Time (s)
Initial Denaturation	95	120
15 cycles	95	30
	56	30
	72	120
Final Elongation	72	600
Hold	4	∞

Supplemental Table S27: Reaction conditions for Phusion PCR

Step	Temp (°C)	Time (s)
Initial Denaturation	98	30
30 cycles	98	10
	55-65	20
	72	120
Final Elongation	72	300
Hold	4	∞

Supplemental Table S28: Reaction conditions for Colony PCR

Step	Temp (°C)	Time (s)
Initial Denaturation	95	120
35 cycles	95	30
	60	30
	72	120
Final Elongation	72	420
Hold	4	∞

Primer

Supplemental Table S29: 5’- and 3’-primer sequences used for site saturation mutagenesis.

position	5'-3'	3'-5'
D236	XAATGTCGGAATTNNSAGTCACTTCGAATTCGCCATTGATAAC	GACTSNNAATTCCGACATTGATCAATC
W367	CAACTACTTCACANNSACCATCAACTCAAGCAGTTTACTCCTC	TGGTSNNTGTGAAGTAGTTGGTGAATGC
I424	CACCGGCTTCGGCNNSTGGCATCCTATCCATCTCCACGGCC	GCCASNNGCCGAAGCCGGTGAGATCTTC

Supplemental Table S30: 5’- and 3’-primer sequences for site directed and combinatorial mutagenesis.

position	5'-3'	3'-5'
I424M	CGGCTTCGGCATGTGGCATCCTATCCATCTC	ATGCCGAAGCCGGTGAGATC
I424G	CGGCTTCGGCGGTTGGCATCCTATCCATCTC	XXGCCGAAGCCGGTGAGATCTTC
D236E	AATGTCGGAATTGAAAGTCACTTCGAATTCGC	AATTCCGACATTGATCAATC
A180D	CGAAATTTGGGACACCGATAGACTCGGCGCTCC	GGTGTCCCAAATTTCGAAAAC
I491N	CTTCTTCATTGTCATAACGCATGGCACGCATCTGAG	ATGACAATGAAGAAGCCATG
L513F	CATCTGAGGGGTTCGCAATGC	CCCTCAGATGCGTGCCATGC
L499M	CATCTGAGGGGATGGCAATGC	CCCTCAGATGCGTGCCATGC
I424S	ACCGGCTTCGGCTCTTGGCATCCTATCCATCTC	GCCGAAGCCGGTGAGATCTTC
I270S	CACTTCTCATTGGCAGCGGTCAAAGATACGATGTCATTG	GCCAATGAGAAGTGTGTCGG

Media

Supplemental Table S31: Media abbreviations

Medium	Abbreviation
LB ± Zeo	Luria-Broth (± antibiotic zeocin)
YPD ± Zeo	Yeast Extract-Peptone-Dextrose (± antibiotic Zeocin)
BYPD	Buffered Yeast Extract-Peptone-Dextrose (± antibiotic Zeocin)
BYPG	Buffered Yeast Extract-Peptone-Glucose (± antibiotic Zeocin)
YNB	Yeast Nitrogen Base
BMG + AC	Buffered minimal glycerol (+ ABTS and Cu[II]So_4)
FBSM	Fermentation basalt salt medium
PTM	Pichia trace metal salts

Supplemental Table S32: Media composition

Medium	Composition
LB ± Zeo	10 g L^{-1} peptone from casein, 5 g L^{-1} yeast extract, 2.5 g L^{-1} NaCl, 15 g L^{-1}Agar Agar, 25 mg L^{-1} zeocin (100 mg mL^{-1})
YPD ± Zeo	20 g L^{-1}peptone from casein, 5 g L^{-1} yeast extract, 0.4 g L^{-1} glucose, 15 g L^{-1} Agar, 100 mg L^{-1} zeocin (100 mg L^{-1})
BYPD	20 g L^{-1}peptone from casein, 5 g L^{-1} yeast extract, 0.4 g L^{-1} glucose, 100 mM KPO_4 buffer pH 5.0
BYPG	20 g L^{-1}peptone from casein, 5 g L^{-1} yeast extract, 4% (200 mL L^{-1}) glycerol (20%), 100 mM KPO_4 buffer pH 5.0
10x YNB	36 g L^{-1} yeast nitrogen base without amino acids and ammonia sulphate, 100 g L^{-1} $(NH_4)2SO_4$
BMG + AC	100 mL L^{-1} 10x YNB, 2 mL L^{-1} biotin (200 mg L^{-1}), 0.2 mM ABTS, 15 g L^{-1} Agar, 1% glycerol, 0.1 mM $CuSO_4$, 100 mM KPO_4 buffer of desired pH
FBSM	26.7 mL L^{-1} HPO_4 (85%), 0.93 g L^{-1} $CaSO_4$, 18.2 g L^{-1} KSO_4, 14.9 g L^{-1} $MgSO_4$, 4.1 g L^{-1} KOH, 40 g L^{-1} glycerol
PTM	6.0 g L^{-1} $CuSO_4{\cdot}5H_2O$, 0.08 g L^{-1} NaCI, 3.0 g L^{-1} $MnSO_4{\cdot}H_2O$, 0.2 g L^{-1} $Na_2MoO_4{\cdot}2H_2O$, 0.2 g L^{-1} H_3BO_3, 0.5 g L^{-1} $CoCl_2$, 20 g L^{-1} $ZnCl_2$, 65 g L^{-1} $FeSO_4{\cdot}7H_2O$, 5 mL L^{-1} H_2SO_4

Supplemental Figures

Introduction

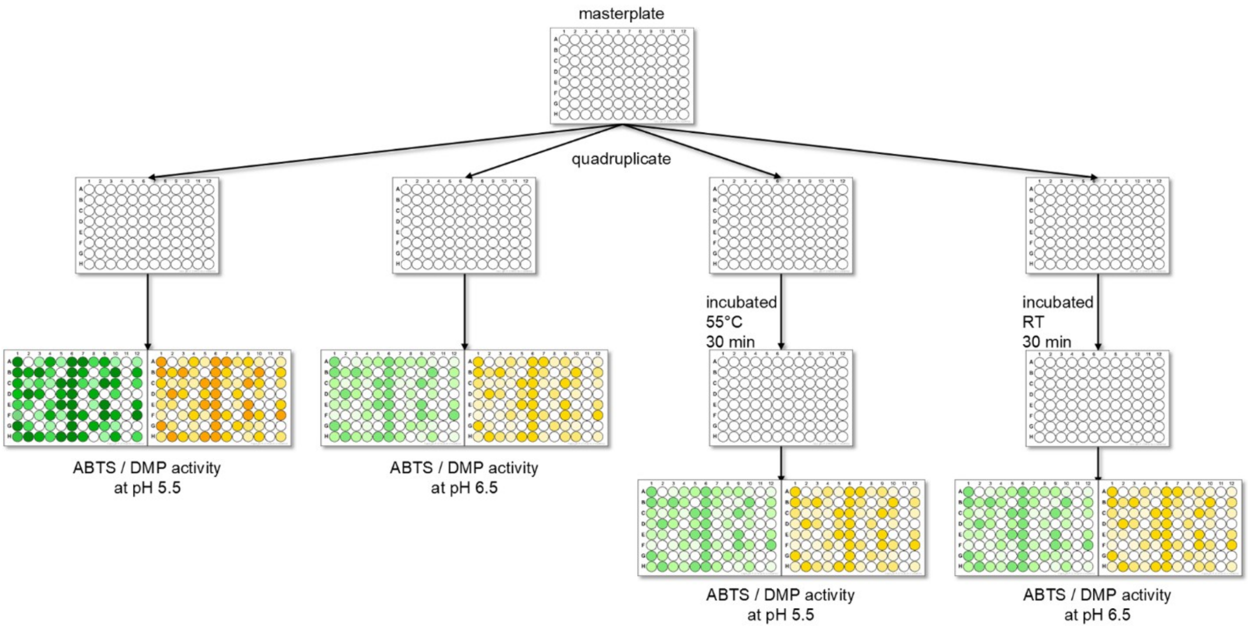

Supplemental Figure S1: Scheme of the differential screening process.

pH profiles

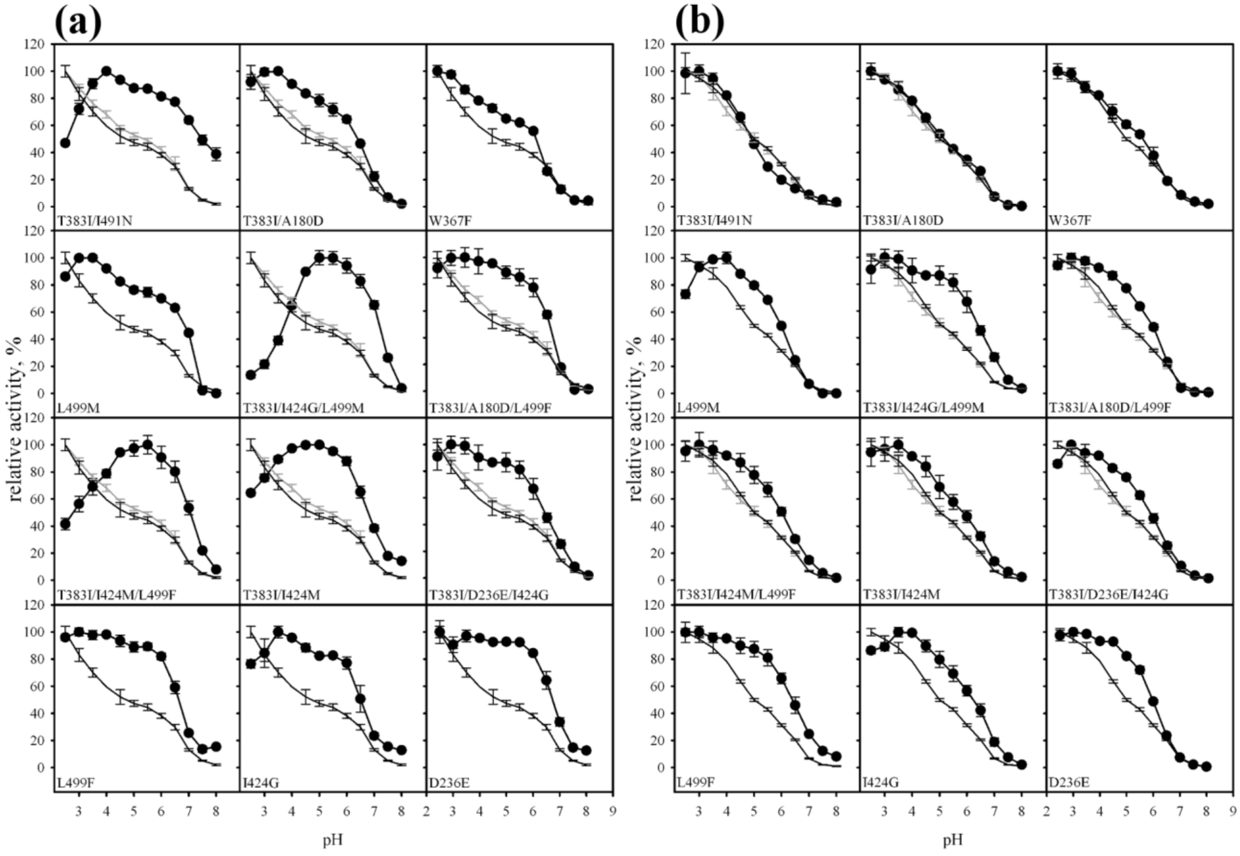

Supplemental Figure S2: pH-profiles by relative activity of depicted *Ba*L variants (black circles) with (a) DMP and (b) ABTS as substrate. Black solid line, *Ba*L wild-type. Grey solid line, *Ba*L T383I.

Apparent steady-state kinetic constants

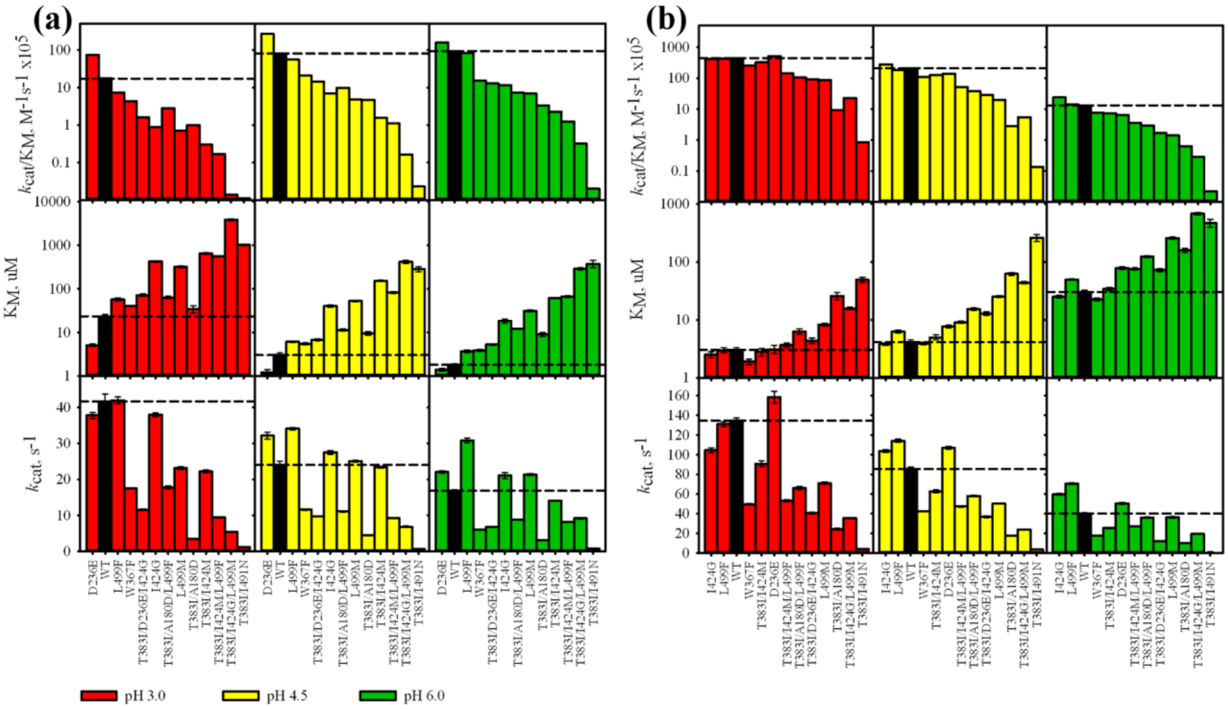

Supplemental Figure S3: Apparent steady-state kinetic constants of *Ba*L variants. The catalytic efficiency k_{cat}/K_M (upper panels), the Michaelis constant K_M (middle panels) and the catalytic constant k_{cat} (lower panels) were determined for the substrates DMP (a) and ABTS (b) at pH 3.0 (red), 4.5 (yellow) and 6.0 (green). Corresponding value for wild-type *Ba*L (black, - - -). The variants are arranged with respect to their k_{cat}/K_M at pH 6.0 from highest to lowest, and the resulting order was also used for pH 3.0 and 4.5.

Multiple Sequence Alignment

A HRPL B.aclada Lac	---SAIPAV-------RSTLTPRQ-------NTTA-----------SCANSATSRSCUGE--YSIDTNWYD---VTP-TG	[80]
A M.mycetomatis Lac	-----SPAK-------PDPVVGLLPRQTIVPGGRP-----------CGQHGPTNRGCUKN-NWNISTDYEM---DTPPAF	[80]
A S.minor Lac	---SAIPAI-------RSPATSHQ-------KVTA-----------SCAHSATSRSCUGA--YSIDTDWYD---VIPHTG	[80]
A M.fructigena Lac	---SAIPAT-------SSPIFPRQ-------NTTSNI-------TPTCAHSSTSRSCUGA--YSIDTNWYD---VTP-TG	[80]
A P.liquidambaris Lac	---MSLPSLQ----DLTSNLMPRQ---------------------SNCQNSPTSRGCUGN--YSIDTNYYD---VFPDTG	[80]
A B.cinerea Lac	---LPTAEV------IPSALEERQ----------------------SCANTATTRSCUGQ--YSASTNSYT---TVPKTG	[80]
A Y.lipolytica Lac	---A	[80]
A C.platani Lac	---------------VPTTISQEV-------GKRA-----------STCNTAEDRACUSD-GFSIDTNYME---SFPDTG	[80]
A M.albomyces Lac	-----E--PTCNTPSNRACUSD-GFDINTDYEV---STPDTG	[80]
A T.arenaria Lac	----APPANP----VQRDMLQVLE-------ARQS----------GPTCNTPSNRACUTN-GFDINTDYEV---STPNTG	[80]
A N.crassa Lac	----APAPGT----EGVNLLTPVD-------KRQDSQAERYGGGGGGGCNSPTNRQCUSP-GFNINTDYEL---GTPNTG	[80]
A S.fimicola Lac	----APALGN----DRVNLLTPVD-------KRQDFVAPGFGGGFGGNCNSPTNRQCUSL-GFNINTDFEL---GTPTTG	[80]
A C.parasitica Lac	-----APSL----------LHPLE-------PRQQ-----------PNCNTASNRACUISGSYDITTDYEV---KTPLTG	[80]
A V.mali Lac	----AVPSL-------ANRLEPIL-------ARQQ-----------ESCNTATDRSCUTD-GFDINTDYEI---STPLTG	[80]
B HRPL T.sanguinea Lac	---A	[80]
B HRPL P.coccineus Lac	---A	[80]
B HRPL T.versicolor Lac	---A	[80]
B HRPL T.hirsuta Lac	---A	[80]
B HRPL T.villosa Lac	---G	[80]
B HRPL PM1 Lac	---S	[80]
B HRPL P.ostreatus Lac	---A	[80]
B C.cinereus Lac	--AI	[80]
B P.cinnabarinus Lac	---A	[80]
B C.maxima Lac	---G	[80]
B C.gallica Lac	---A	[80]
B T.trogii Lac	---A	[80]
B L.tigrinus Lac	---A	[80]
B C.zonatus Lac	---A	[80]
B S.murashkinsky Lac	--AQ	[80]
B A.faginea Lac	---A	[80]
B R.lignosus Lac	--AT	[80]
P T.vernicifluum Lac	---V	[80]
P B.napus Lac	--	[80]
P P.trichocarpa Lac	--	[80]
P C.clementina Lac	--	[80]
P M.notabilis Lac	--	[80]
P T.cacao Lac	--	[80]
P M.truncatula Lac	--	[80]
P P.persica Lac	--	[80]
P E.grandis Lac	--	[80]
P G.max Lac	--	[80]
P A.thaliana Lac	---S	[80]
P P.sativum AscOx	---A	[80]
P M.truncatula L-AscOx	---K	[80]
P G.soja L-AscOx	---G	[80]
P C.pepo L-AscOx	---S	[80]
P C.melo AscOx	MAKVADKPFFPKPFLVFFVLSIIFGFGITLSEAGF--P	[80]
P Z.marina L-AscOx	---IWK---R	[80]
Ba B.halodurans alkLac	--DDHSEMDH-GNNQQ-DEQN---EGTFED	[80]
Ba B.amyloliquefacien Cot	---MALEKF---ADE-LPIIETLQPQKKSD	[80]
Ba B.subtilis CotA	---MTLEKF---VDA-LPIPDTLKPVQQSK	[80]
Ba B.subtilis CO	---MTLEKF---VDA-LPIPDTLKPVQQSK	[80]
Ba B.pumilus CotA	---MNLEKF---VDE-LPIPEVAEPVKKNP	[80]
Ba B.sonorensis CotA	---MKLEKF---VDQ-LPIPKVIKPHTKSK	[80]
Ba B.subtilis outerpCotA	-MKMTLEKF---VDA-LPIPDTLKPVQQSK	[80]
Ba B.vallismortis Lac	---MTLEKF---VDA-LPIPDTLKPVQQSK	[80]
Ba T.thermophilus Lac	--GPS-FPEPK---VVRSQG	[80]
Ba R.bacterium Lac	---MAT--ASATAILPM---PTFAAE	[80]

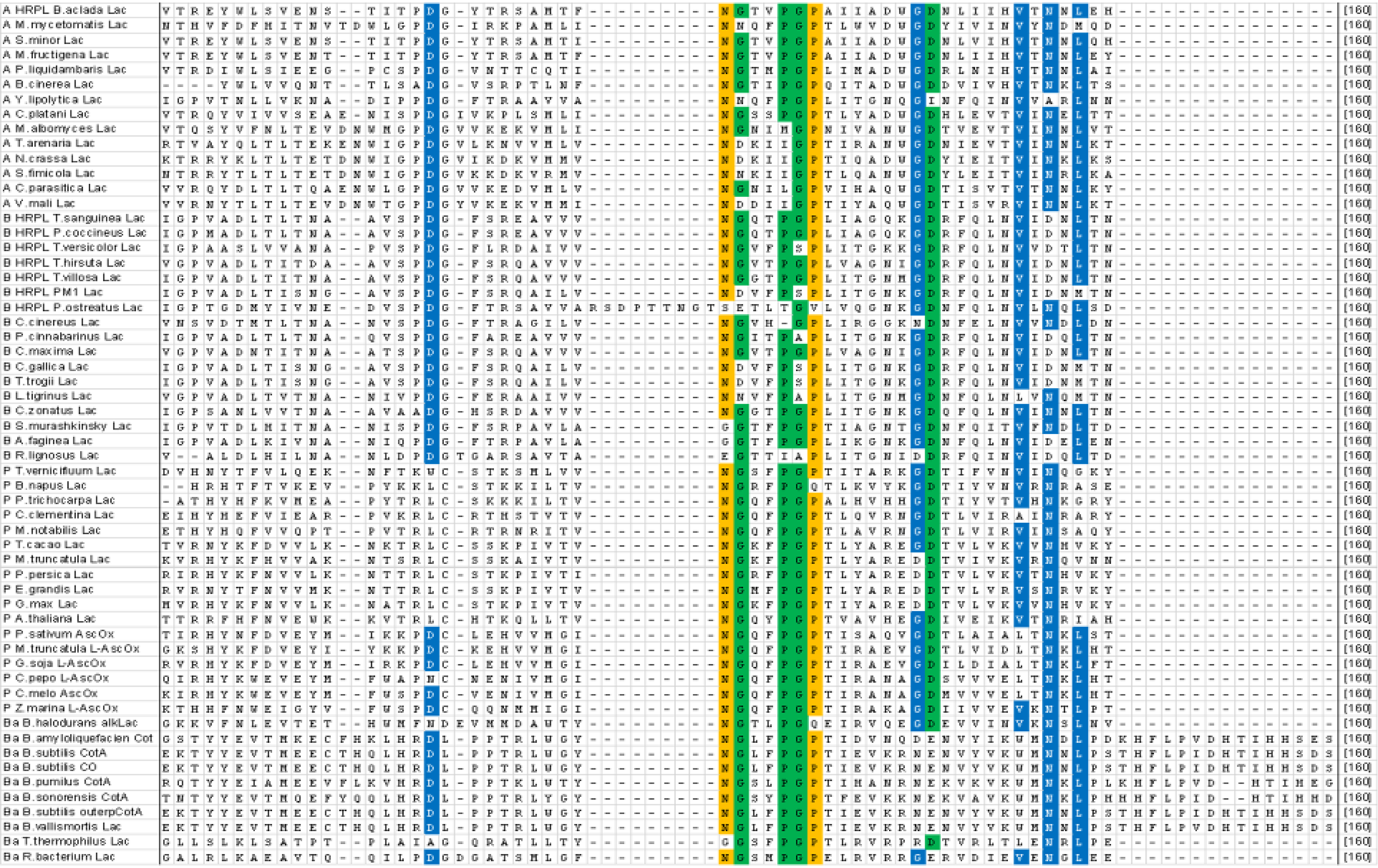
A HRPL B.aclada Lac
A M.mycetomatis Lac
A S.minor Lac
A M.fructigena Lac
A P.liquidambaris Lac
A B.cinerea Lac
A Y.lipolytica Lac
A C.platani Lac
A M.albomyces Lac
A T.arenaria Lac
A N.crassa Lac
A S.fimicola Lac
A C.parasitica Lac
A V.mali Lac
B HRPL T.sanguinea Lac
B HRPL P.coccineus Lac
B HRPL T.versicolor Lac
B HRPL T.hirsuta Lac
B HRPL T.villosa Lac
B HRPL PM1 Lac
B HRPL P.ostreatus Lac
B C.cinereus Lac
B P.cinnabarinus Lac
B C.maxima Lac
B C.gallica Lac
B T.trogii Lac
B L.tigrinus Lac
B C.zonatus Lac
B S.murashkinsky Lac
B A.faginea Lac
B R.lignosus Lac
P T.vernicifluum Lac
P B.napus Lac
P P.trichocarpa Lac
P C.clementina Lac
P M.notabilis Lac
P T.cacao Lac
P M.truncatula Lac
P P.persica Lac
P E.grandis Lac
P G.max Lac
P A.thaliana Lac
P P.sativum AscOx
P M.truncatula L-AscOx
P G.soja L-AscOx
P C.pepo L-AscOx
P C.melo AscOx
P Z.marina L-AscOx
Ba B.halodurans alkLac
Ba B.amyloliquefacien Cot
Ba B.subtilis CotA
Ba B.subtilis CO
Ba B.pumilus CotA
Ba B.sonorensis CotA
Ba B.subtilis outerpCotA
Ba B.vallismortis Lac
Ba T.thermophilus Lac
Ba R.bacterium Lac
[160]

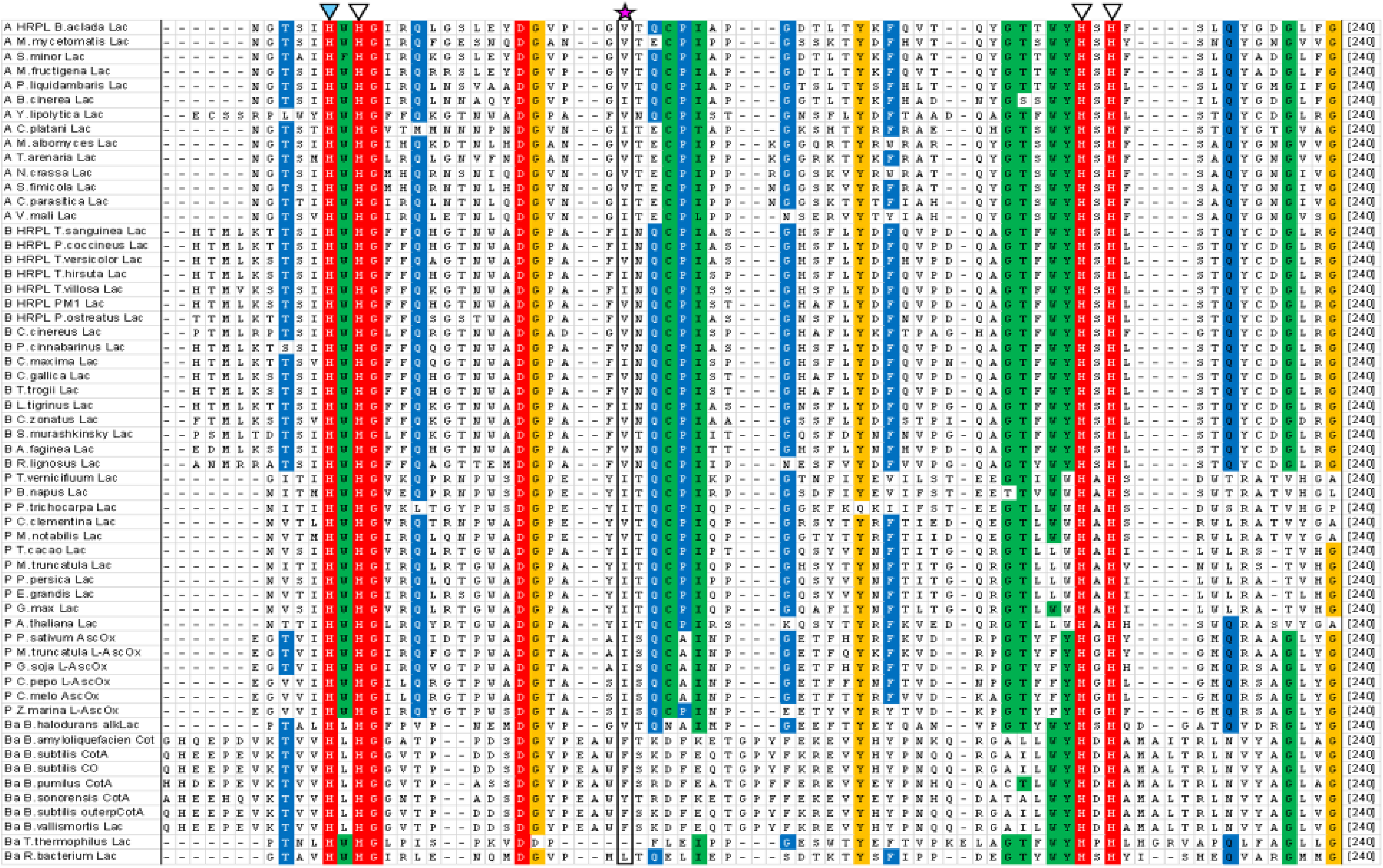
A HRPL B.aclada Lac
A M.mycetomatis Lac
A S.minor Lac
A M.fructigena Lac
A P.liquidambaris Lac
A B.cinerea Lac
A Y.lipolytica Lac
A C.platani Lac
A M.albomyces Lac
A T.arenaria Lac
A N.crassa Lac
A S.fimicola Lac
A C.parasitica Lac
A V.mali Lac
B HRPL T.sanguinea Lac
B HRPL P.coccineus Lac
B HRPL T.versicolor Lac
B HRPL T.hirsuta Lac
B HRPL T.villosa Lac
B HRPL PM1 Lac
B HRPL P.ostreatus Lac
B C.cinereus Lac
B P.cinnabarinus Lac
B C.maxima Lac
B C.gallica Lac
B T.trogii Lac
B L.tigrinus Lac
B C.zonatus Lac
B S.murashkinsky Lac
B A.faginea Lac
B R.lignosus Lac
P T.vernicifluum Lac
P B.napus Lac
P P.trichocarpa Lac
P C.clementina Lac
P M.notabilis Lac
P T.cacao Lac
P M.truncatula Lac
P P.persica Lac
P E.grandis Lac
P G.max Lac
P A.thaliana Lac
P P.sativum AscOx
P M.truncatula L-AscOx
P G.soja L-AscOx
P C.pepo L-AscOx
P C.melo AscOx
P Z.marina L-AscOx
Ba B.halodurans alkLac
Ba B.amyloliquefacien Cot
Ba B.subtilis CotA
Ba B.subtilis CO
Ba B.pumilus CotA
Ba B.sonorensis CotA
Ba B.subtilis outerpCotA
Ba B.vallismortis Lac
Ba T.thermophilus Lac
Ba R.bacterium Lac

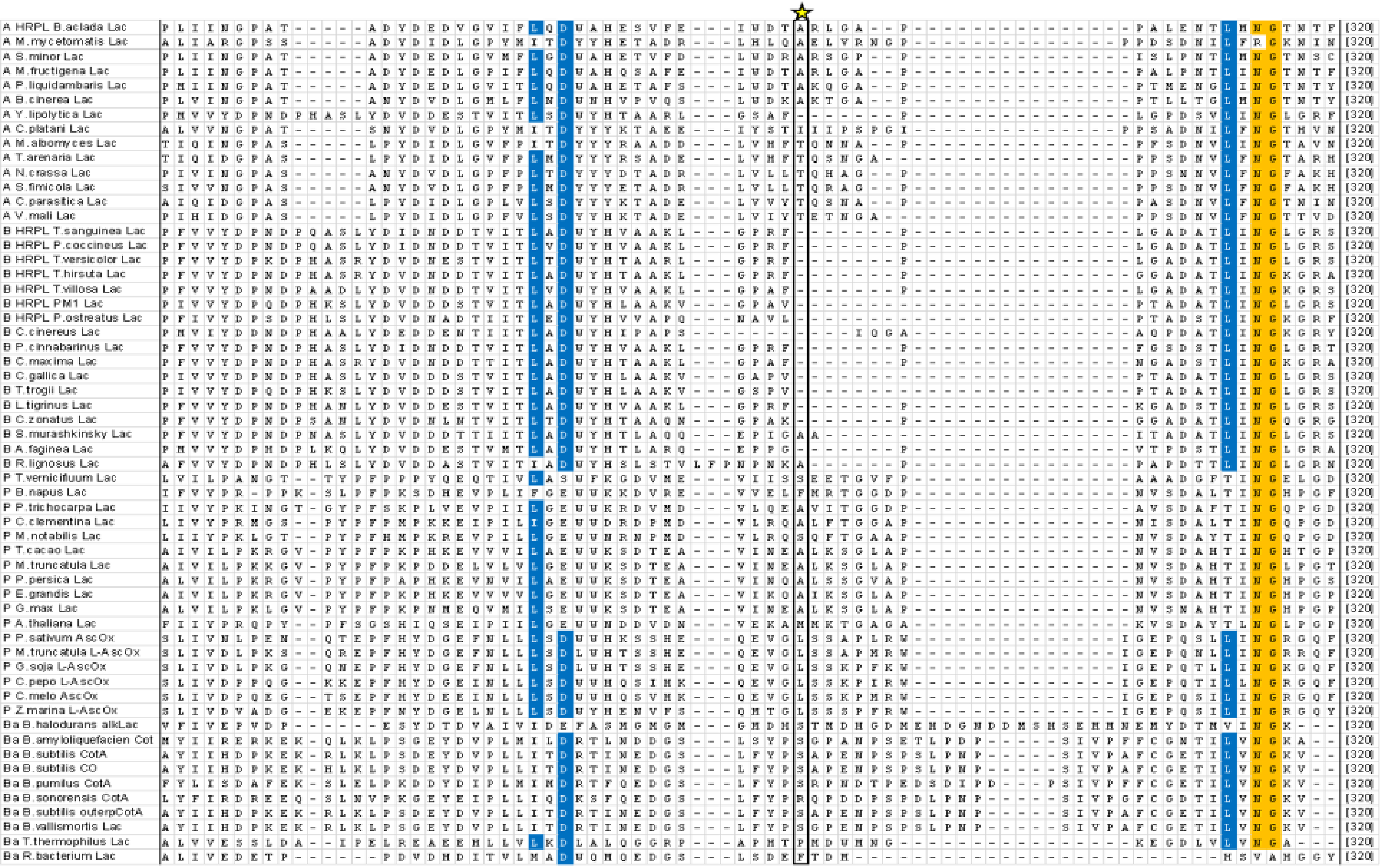
A HRPL B.aclada Lac
A M.mycetomatis Lac
A S.minor Lac
A M.fructigena Lac
A P.liquidambaris Lac
A B.cinerea Lac
A Y.lipolytica Lac
A C.platani Lac
A M.albomyces Lac
A T.arenaria Lac
A N.crassa Lac
A S.fimicola Lac
A C.parasitica Lac
A V.mali Lac
B HRPL T.sanguinea Lac
B HRPL P.coccineus Lac
B HRPL T.versicolor Lac
B HRPL T.hirsuta Lac
B HRPL T.villosa Lac
B HRPL PM1 Lac
B HRPL P.ostreatus Lac
B C.cinereus Lac
B P.cinnabarinus Lac
B C.maxima Lac
B C.gallica Lac
B T.trogii Lac
B L.tigrinus Lac
B C.zonatus Lac
B S.murashkinsky Lac
B A.faginea Lac
B R.lignosus Lac
P T.vernicifluum Lac
P B.napus Lac
P P.trichocarpa Lac
P C.clementina Lac
P M.notabilis Lac
P T.cacao Lac
P M.truncatula Lac
P P.persica Lac
P E.grandis Lac
P G.max Lac
P A.thaliana Lac
P P.sativum AscOx
P M.truncatula L-AscOx
P G.soja L-AscOx
P C.pepo L-AscOx
P C.melo AscOx
P Z.marina L-AscOx
Ba B.halodurans alkLac
Ba B.amyloliquefacien Cot
Ba B.subtilis CotA
Ba B.subtilis CO
Ba B.pumilus CotA
Ba B.sonorensis CotA
Ba B.subtilis outerpCotA
Ba B.vallismortis Lac
Ba T.thermophilus Lac
Ba R.bacterium Lac

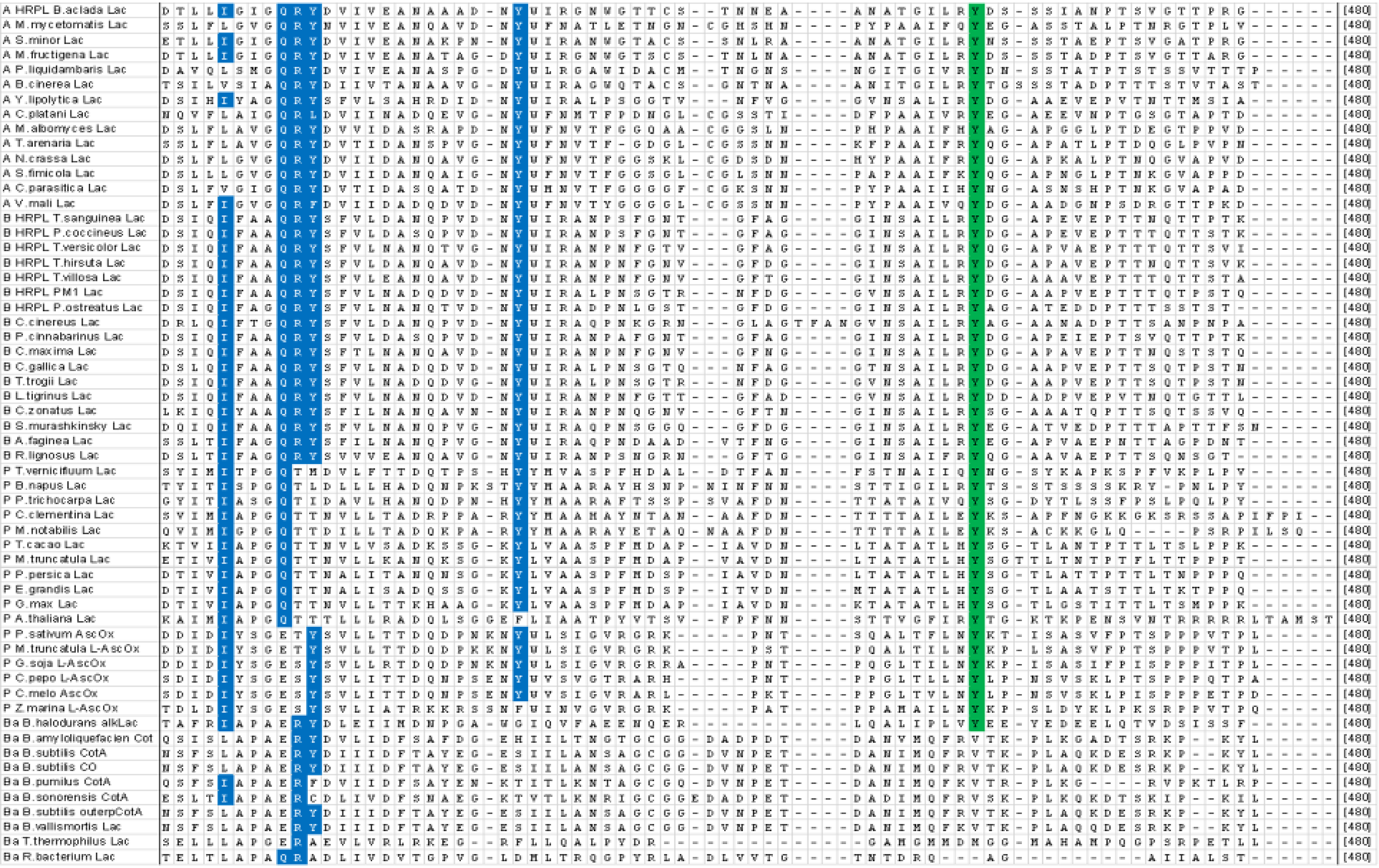
A HRPL B.aclada Lac
A M.mycetomatis Lac
A S.minor Lac
A M.fructigena Lac
A P.liquidambaris Lac
A B.cinerea Lac
A Y.lipolytica Lac
A C.platani Lac
A M.albomyces Lac
A T.arenaria Lac
A N.crassa Lac
A S.fimicola Lac
A C.parasitica Lac
A V.mali Lac
B HRPL T.sanguinea Lac
B HRPL P.coccineus Lac
B HRPL T.versicolor Lac
B HRPL T.hirsuta Lac
B HRPL T.villosa Lac
B HRPL PM1 Lac
B HRPL P.ostreatus Lac
B C.cinereus Lac
B P.cinnabarinus Lac
B C.maxima Lac
B C.gallica Lac
B T.trogii Lac
B L.tigrinus Lac
B C.zonatus Lac
B S.murashkinsky Lac
B A.faginea Lac
B R.lignosus Lac
P T.vernicifluum Lac
P B.napus Lac
P P.trichocarpa Lac
P C.clementina Lac
P M.notabilis Lac
P T.cacao Lac
P M.truncatula Lac
P P.persica Lac
P E.grandis Lac
P G.max Lac
P A.thaliana Lac
P P.sativum AscOx
P M.truncatula L-AscOx
P G.soja L-AscOx
P C.pepo L-AscOx
P C.melo AscOx
P Z.marina L-AscOx
Ba B.halodurans alkLac
Ba B.amyloliquefacien Cot
Ba B.subtilis CotA
Ba B.subtilis CO
Ba B.pumilus CotA
Ba B.sonorensis CotA
Ba B.subtilis outerpCotA
Ba B.vallismortis Lac
Ba T.thermophilus Lac
Ba R.bacterium Lac
[480]

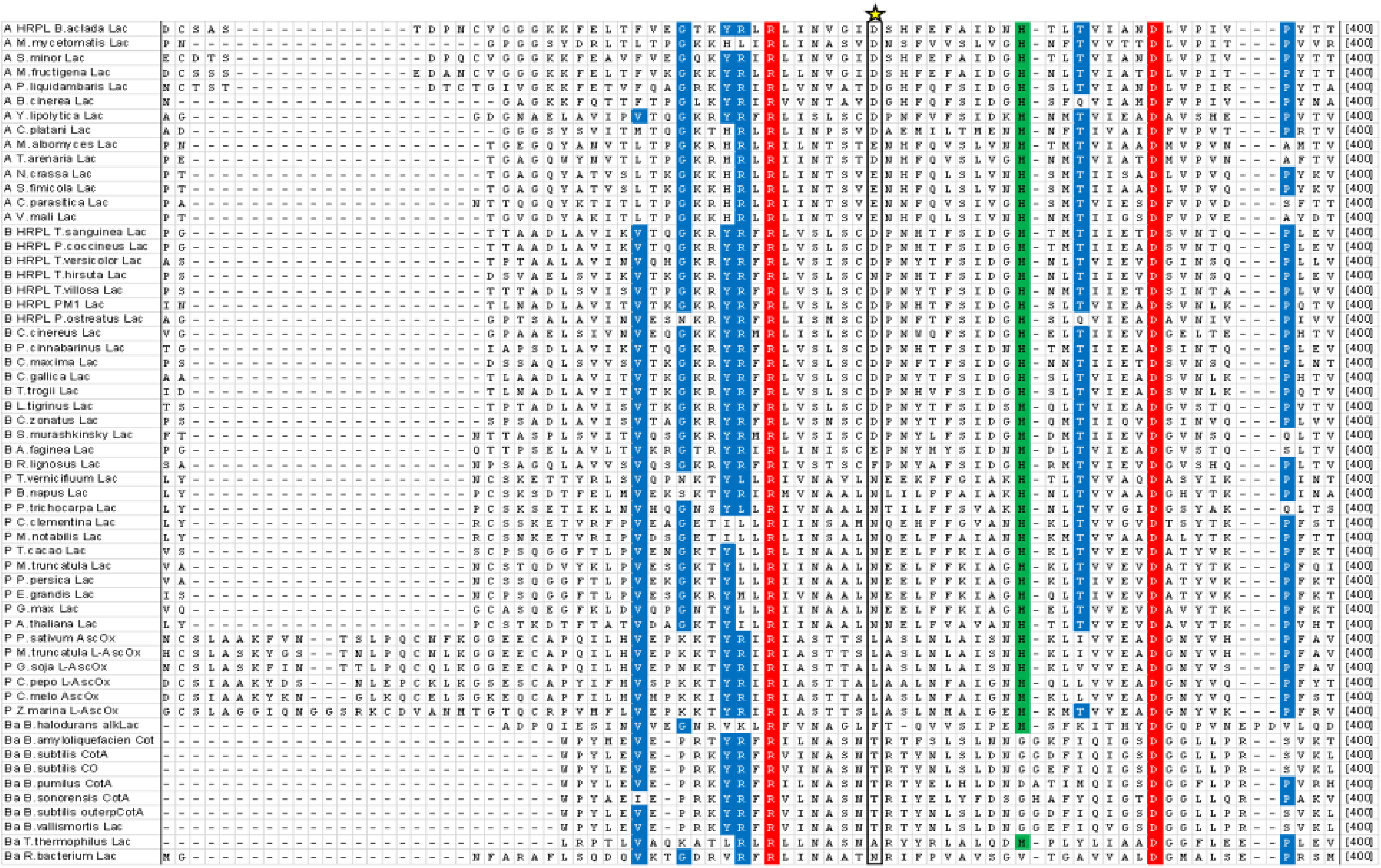
A HRPL B.aclada Lac
A M.mycetomatis Lac
A S.minor Lac
A M.fructigena Lac
A P.liquidambaris Lac
A B.cinerea Lac
A Y.lipolytica Lac
A C.platani Lac
A M.albomyces Lac
A T.arenaria Lac
A N.crassa Lac
A S.fimicola Lac
A C.parasitica Lac
A V.mali Lac
B HRPL T.sanguinea Lac
B HRPL P.coccineus Lac
B HRPL T.versicolor Lac
B HRPL T.hirsuta Lac
B HRPL T.villosa Lac
B HRPL PM1 Lac
B HRPL P.ostreatus Lac
B C.cinereus Lac
B P.cinnabarinus Lac
B C.maxima Lac
B C.gallica Lac
B T.trogii Lac
B L.tigrinus Lac
B C.zonatus Lac
B S.murashkinsky Lac
B A.faginea Lac
B R.lignosus Lac
P T.vernicifluum Lac
P B.napus Lac
P P.trichocarpa Lac
P C.clementina Lac
P M.notabilis Lac
P T.cacao Lac
P M.truncatula Lac
P P.persica Lac
P E.grandis Lac
P G.max Lac
P A.thaliana Lac
P P.sativum AscOx
P M.truncatula L-AscOx
P G.soja L-AscOx
P C.pepo L-AscOx
P C.melo AscOx
P Z.marina L-AscOx
Ba B.halodurans alkLac
Ba B.amyloliquefacien Cot
Ba B.subtilis CotA
Ba B.subtilis CO
Ba B.pumilus CotA
Ba B.sonorensis CotA
Ba B.subtilis outerpCotA
Ba B.vallismortis Lac
Ba T.thermophilus Lac
Ba R.bacterium Lac
[400]

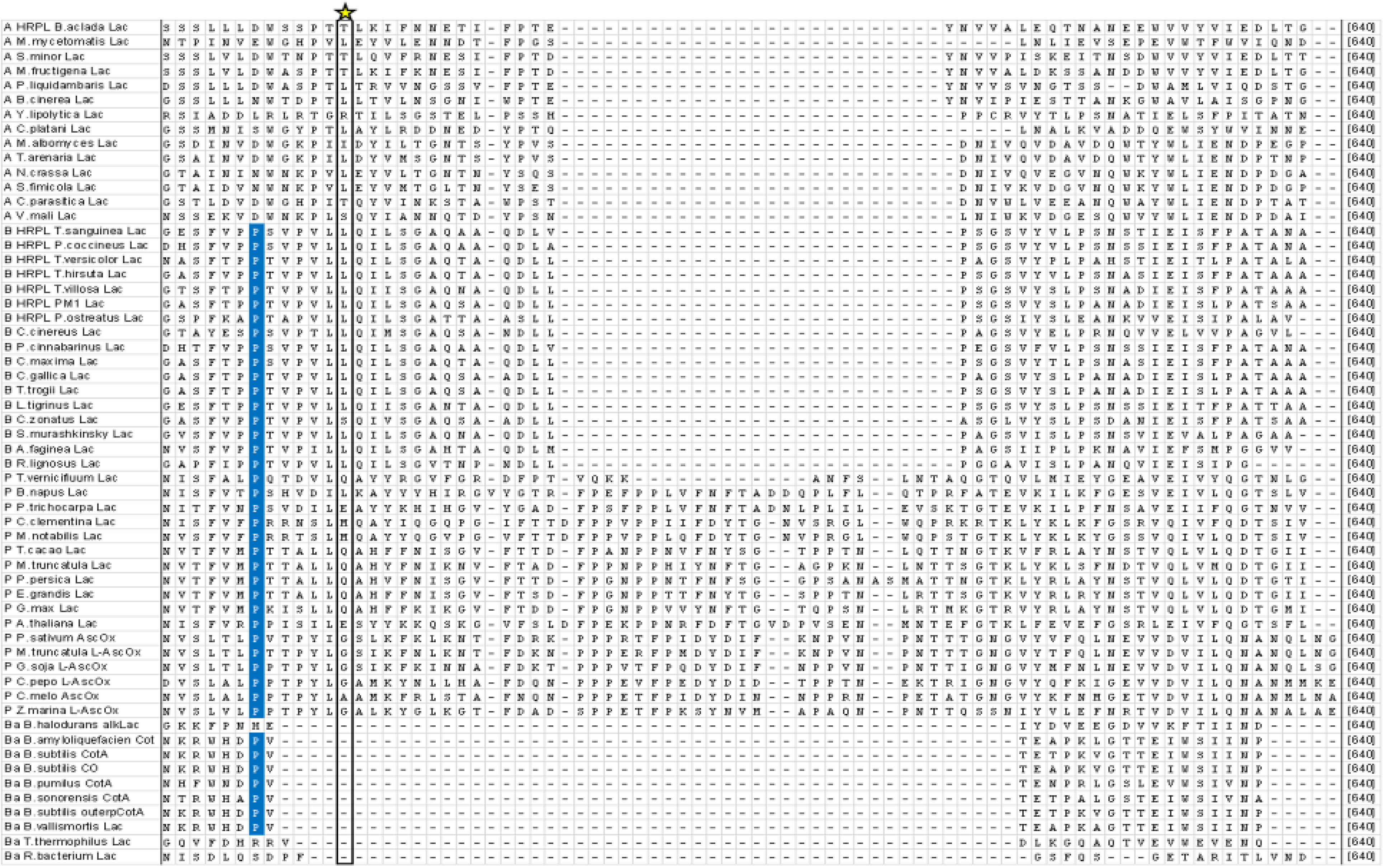

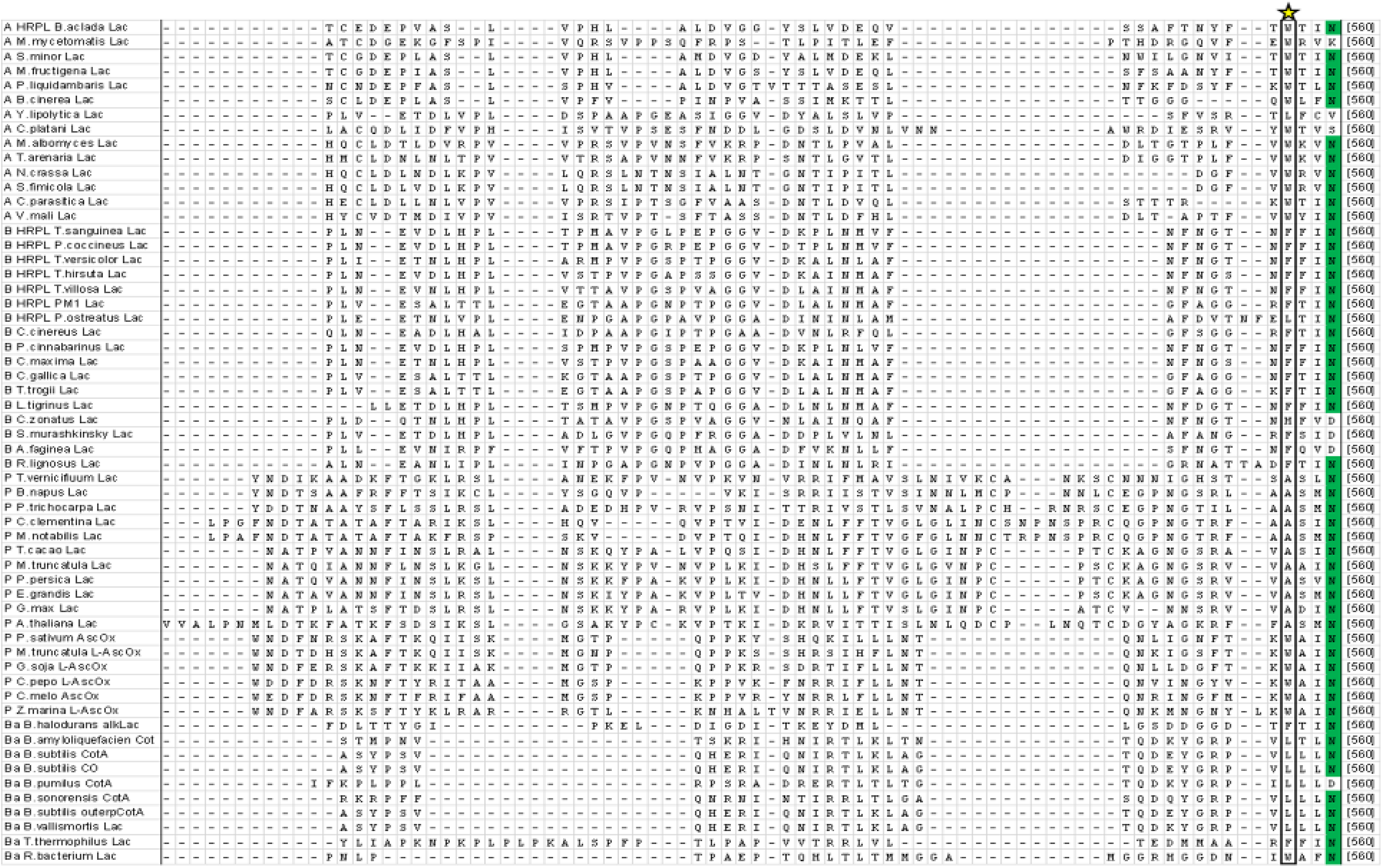

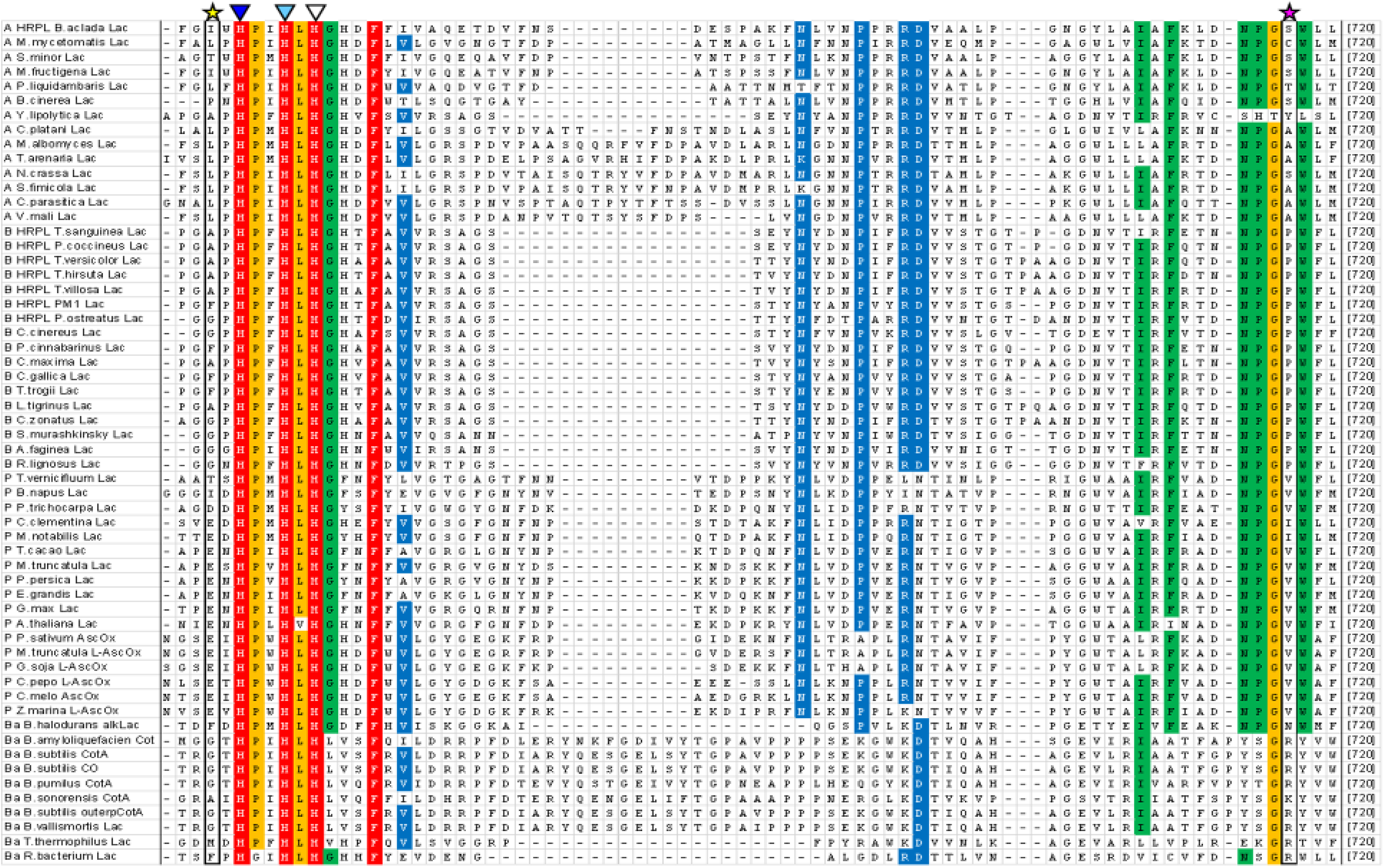
A HRPL B.aclada Lac
A M.mycetomatis Lac
A S.minor Lac
A M.fructigena Lac
A P.liquidambaris Lac
A B.cinerea Lac
A Y.lipolytica Lac
A C.platani Lac
A M.albomyces Lac
A T.arenaria Lac
A N.crassa Lac
A S.fimicola Lac
A C.parasitica Lac
A V.mali Lac
B HRPL T.sanguinea Lac
B HRPL P.coccineus Lac
B HRPL T.versicolor Lac
B HRPL T.hirsuta Lac
B HRPL T.villosa Lac
B HRPL PM1 Lac
B HRPL P.ostreatus Lac
B C.cinereus Lac
B P.cinnabarinus Lac
B C.maxima Lac
B C.gallica Lac
B T.trogii Lac
B L.tigrinus Lac
B C.zonatus Lac
B S.murashkinsky Lac
B A.faginea Lac
B R.lignosus Lac
P T.vernicifluum Lac
P B.napus Lac
P P.trichocarpa Lac
P C.clementina Lac
P M.notabilis Lac
P T.cacao Lac
P M.truncatula Lac
P P.persica Lac
P E.grandis Lac
P G.max Lac
P A.thaliana Lac
P P.sativum AscOx
P M.truncatula L-AscOx
P G.soja L-AscOx
P C.pepo L-AscOx
P C.melo AscOx
P Z.marina L-AscOx
Ba B.halodurans alkLac
Ba B.amyloliquefacien Cot
Ba B.subtilis CotA
Ba B.subtilis CO
Ba B.pumilus CotA
Ba B.sonorensis CotA
Ba B.subtilis outerpCotA
Ba B.vallismortis Lac
Ba T.thermophilus Lac
Ba R.bacterium Lac
[720]

A HRPL B.aclada Lac	-	-	-	-	-	-	-	-	-	[809]
A M.mycetomatis Lac	M	E	P	A	V	R	R	I	G	[809]
A S.minor Lac	-	-	-	-	-	-	-	-	-	[809]
A M.fructigena Lac	-	-	-	-	-	-	-	-	-	[809]
A P.liquidambaris Lac	-	-	-	-	-	-	-	-	-	[809]
A B.cinerea Lac	-	-	-	-	-	-	-	-	-	[809]
A Y.lipolytica Lac	-	-	-	-	-	-	-	-	-	[809]
A C.platani Lac	-	-	-	-	-	-	-	-	-	[809]
A M.albomyces Lac	-	-	-	-	-	-	-	-	-	[809]
A T.arenaria Lac	-	-	-	-	-	-	-	-	-	[809]
A N.crassa Lac	M	K	W	-	-	-	-	-	-	[809]
A S.fimicola Lac	-	-	-	-	-	-	-	-	-	[809]
A C.parasitica Lac	-	-	-	-	-	-	-	-	-	[809]
A V.mali Lac	-	-	-	-	-	-	-	-	-	[809]
B HRPL T.sanguinea Lac	-	-	-	-	-	-	-	-	-	[809]
B HRPL P.coccineus Lac	-	-	-	-	-	-	-	-	-	[809]
B HRPL T.versicolor Lac	-	-	-	-	-	-	-	-	-	[809]
B HRPL T.hirsuta Lac	-	-	-	-	-	-	-	-	-	[809]
B HRPL T.villosa Lac	-	-	-	-	-	-	-	-	-	[809]
B HRPL PM1 Lac	-	-	-	-	-	-	-	-	-	[809]
B HRPL P.ostreatus Lac	-	-	-	-	-	-	-	-	-	[809]
B C.cinereus Lac	-	-	-	-	-	-	-	-	-	[809]
B P.cinnabarinus Lac	-	-	-	-	-	-	-	-	-	[809]
B C.maxima Lac	-	-	-	-	-	-	-	-	-	[809]
B C.gallica Lac	-	-	-	-	-	-	-	-	-	[809]
B T.trogii Lac	-	-	-	-	-	-	-	-	-	[809]
B L.tigrinus Lac	-	-	-	-	-	-	-	-	-	[809]
B C.zonatus Lac	-	-	-	-	-	-	-	-	-	[809]
B S.murashkinsky Lac	R	Q	E	E	R	C	G	I	S	[809]
B A.faginea Lac	-	-	-	-	-	-	-	-	-	[809]
B R.lignosus Lac	-	-	-	-	-	-	-	-	-	[809]
P T.vernicifluum Lac	-	-	-	-	-	-	-	-	-	[809]
P B.napus Lac	-	-	-	-	-	-	-	-	-	[809]
P P.trichocarpa Lac	-	-	-	-	-	-	-	-	-	[809]
P C.clementina Lac	-	-	-	-	-	-	-	-	-	[809]
P M.notabilis Lac	-	-	-	-	-	-	-	-	-	[809]
P T.cacao Lac	-	-	-	-	-	-	-	-	-	[809]
P M.truncatula Lac	-	-	-	-	-	-	-	-	-	[809]
P P.persica Lac	-	-	-	-	-	-	-	-	-	[809]
P E.grandis Lac	-	-	-	-	-	-	-	-	-	[809]
P G.max Lac	-	-	-	-	-	-	-	-	-	[809]
P A.thaliana Lac	-	-	-	-	-	-	-	-	-	[809]
P P.sativum AscOx	-	-	-	-	-	-	-	-	-	[809]
P M.truncatula L-AscOx	-	-	-	-	-	-	-	-	-	[809]
P G.soja L-AscOx	-	-	-	-	-	-	-	-	-	[809]
P C.pepo L-AscOx	-	-	-	-	-	-	-	-	-	[809]
P C.melo AscOx	-	-	-	-	-	-	-	-	-	[809]
P Z.marina L-AscOx	-	-	-	-	-	-	-	-	-	[809]
Ba B.halodurans alkLac	-	-	-	-	-	-	-	-	-	[809]
Ba B.amyloliquefacien Cot	-	-	-	-	-	-	-	-	-	[809]
Ba B.subtilis CotA	-	-	-	-	-	-	-	-	-	[809]
Ba B.subtilis CO	-	-	-	-	-	-	-	-	-	[809]
Ba B.pumilus CotA	-	-	-	-	-	-	-	-	-	[809]
Ba B.sonorensis CotA	-	-	-	-	-	-	-	-	-	[809]
Ba B.subtilis outerpCotA	-	-	-	-	-	-	-	-	-	[809]
Ba B.vallismortis Lac	-	-	-	-	-	-	-	-	-	[809]
Ba T.thermophilus Lac	-	-	-	-	-	-	-	-	-	[809]
Ba R.bacterium Lac	-	-	-	-	-	-	-	-	-	[809]

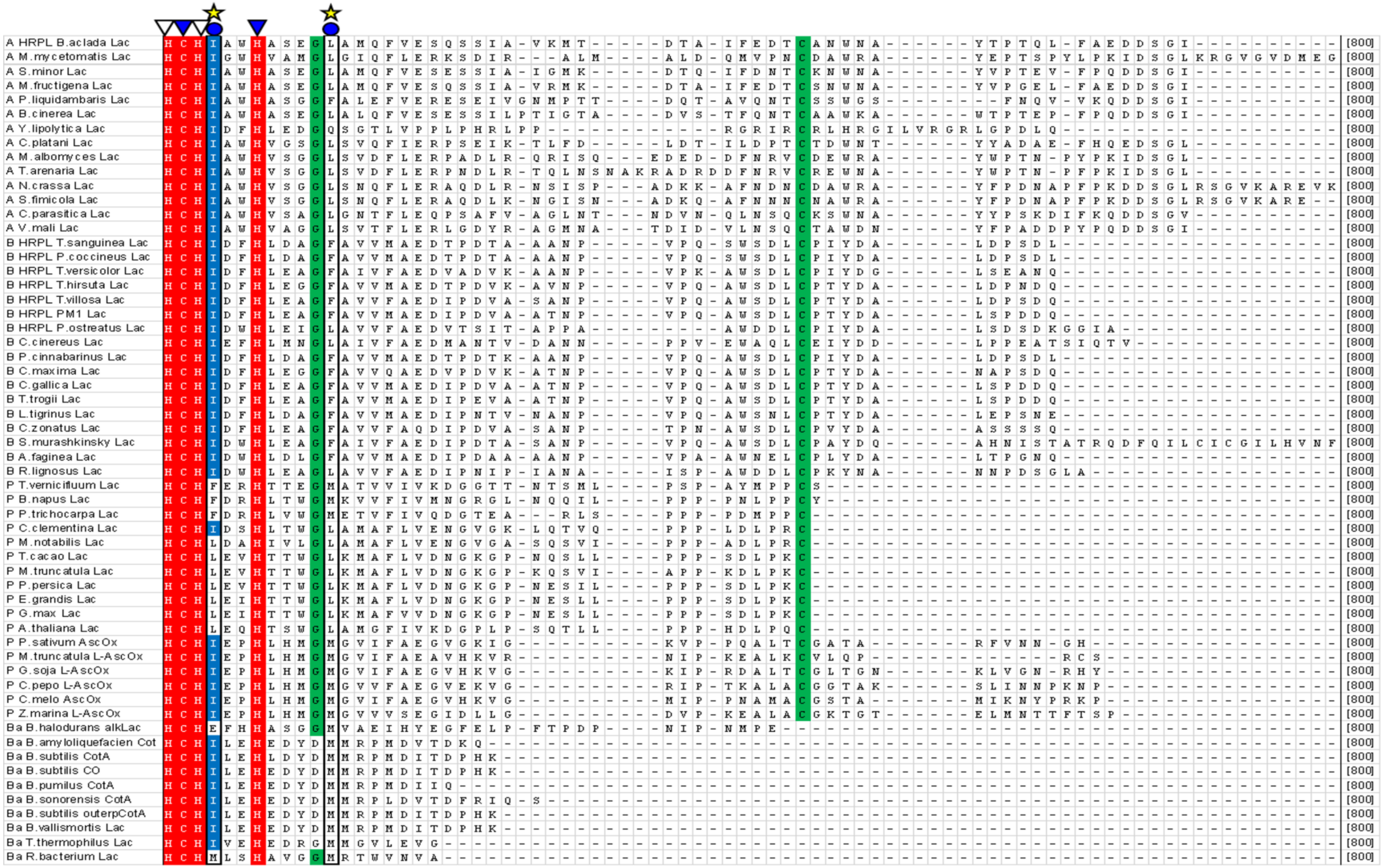
A HRPL B.aclada Lac
A M.mycetomatis Lac
A S.minor Lac
A M.fructigena Lac
A P.liquidambaris Lac
A B.cinerea Lac
A Y.lipolytica Lac
A C.platani Lac
A M.albomyces Lac
A T.arenaria Lac
A N.crassa Lac
A S.fimicola Lac
A C.parasitica Lac
A V.mali Lac
B HRPL T.sanguinea Lac
B HRPL P.coccineus Lac
B HRPL T.versicolor Lac
B HRPL T.hirsuta Lac
B HRPL T.villosa Lac
B HRPL PM1 Lac
B HRPL P.ostreatus Lac
B C.cinereus Lac
B P.cinnabarinus Lac
B C.maxima Lac
B C.gallica Lac
B T.trogii Lac
B L.tigrinus Lac
B C.zonatus Lac
B S.murashkinsky Lac
B A.faginea Lac
B R.lignosus Lac
P T.vernicifluum Lac
P B.napus Lac
P P.trichocarpa Lac
P C.clementina Lac
P M.notabilis Lac
P T.cacao Lac
P M.truncatula Lac
P P.persica Lac
P E.grandis Lac
P G.max Lac
P A.thaliana Lac
P P.sativum AscOx
P M.truncatula L-AscOx
P G.soja L-AscOx
P C.pepo L-AscOx
P C.melo AscOx
P Z.marina L-AscOx
Ba B.halodurans alkLac
Ba B.amyloliquefacien Cot
Ba B.subtilis CotA
Ba B.subtilis CO
Ba B.pumilus CotA
Ba B.sonorensis CotA
Ba B.subtilis outerpCotA
Ba B.vallismortis Lac
Ba T.thermophilus Lac
Ba R.bacterium Lac

Supplemental Figure S4: Multiple sequence and structure alignment of ascomyceteous (A), basidiomyceteous (B), plantal (P) and bacterial (Ba) laccases (Lac), L-ascorbate oxidases (L-AscOx) and spore coat A proteins (CotA). High redox potential laccases are depicted as HRPL in the sequence titles. Red, orange, green and blue highlighted columns show 100, 90, 80 and 70 % conserved residues, respectively. Dark blue triangles: T1-copper coordinating residues; dark blue circles: T1-copper non-coordinating axial ligands; light blue triangles: T2-copper coordinating residues; white triangles: T3-copper coordinating residues; magenta stars: *Ba*L T383 hydrogen bonding positions; yellow stars: *Ba*L positions mutated in this study. The alignment was created by the PROMALS3D online tool (http://prodata.swmed.edu/promals3d/promals3d.php, [7].

Supplemental References

1. Hildén, K., Hakala, T. K. & Lundell, T. Thermotolerant and thermostable laccases. *Biotechnol. Lett.* **31,** 1117–1128 (2009).

2. Osipov, E. *et al.* Effect of the L499M mutation of the ascomycetous *Botrytis aclada* laccase on redox potential and catalytic properties. *Acta Crystallogr. D Biol. Crystallogr.* **70,** 2913–2923 (2014).

3. Bourbonnais, R., Leech, D. & Paice, M. G. Electrochemical analysis of the interactions of laccase mediators with lignin model compounds. *Biochim. Biophys. Acta BBA - Gen. Subj.* **1379,** 381–390 (1998).

4. Jovanovic, S. V., Tosic, M. & Simic, M. G. Use of the Hammett correlation and σ+ for calculation of one-electron redox potentials of antioxidants. *J. Phys. Chem.* **95,** 10824–10827 (1991).

5. Durão, P. *et al.* Proximal mutations at the type 1 copper site of CotA laccase: spectroscopic, redox, kinetic and structural characterization of I494A and L386A mutants. *Biochem. J.* **412,** 339–346 (2008).

6. Sakurai, T. & Kataoka, K. Structure and function of type I copper in multicopper oxidases. *Cell. Mol. Life Sci.* **64,** 2642 (2007).

7. Pei, J., Kim, B.-H. & Grishin, N. V. PROMALS3D: a tool for multiple protein sequence and structure alignments. *Nucleic Acids Res.* **36,** 2295–2300 (2008).

8. Krissinel, E. B. *et al.* The new CCP4 Coordinate Library as a toolkit for the design of coordinate-related applications in protein crystallography. *Acta Crystallogr. D Biol. Crystallogr.* **60,** 2250–2255 (2004).

9. Krissinel, E. & Henrick, K. Secondary-structure matching (SSM), a new tool for fast protein structure alignment in three dimensions. *Acta Crystallogr. D Biol. Crystallogr.* **60,** 2256–2268 (2004).

10. Krissinel, E. B. & Henrick, K. Common subgraph isomorphism detection by backtracking search. *Softw. Pract. Exp.* **34,** 591–607 (2004).

11. Krissinel, E. & Henrick, K. Multiple Alignment of Protein Structures in Three Dimensions. in *Computational Life Sciences* (eds. Berthold, M. R., Glen, R. C., Diederichs, K., Kohlbacher, O. & Fischer, I.) 67–78 (Springer Berlin Heidelberg, 2005). doi:10.1007/11560500_7
